# Mulberry Leaf Compounds and Gut Microbiota in Alzheimer’s Disease and Diabetes: A Study Using Network Pharmacology, Molecular Dynamics Simulation, and Cellular Assays

**DOI:** 10.3390/ijms25074062

**Published:** 2024-04-05

**Authors:** Xue Bai, Xinyi Zhao, Kaifeng Liu, Xiaotang Yang, Qizheng He, Yilin Gao, Wannan Li, Weiwei Han

**Affiliations:** 1 Key Laboratory for Molecular Enzymology and Engineering of Ministry of Education, School of Life Sciences, Jilin University, Changchun 130012, China; baix23@mails.jlu.edu.cn (X.B.); xinyiz21@mails.jlu.edu.cn (X.Z.); liukf1220@mails.jlu.edu.cn (K.L.); yangxt22@mails.jlu.edu.cn (X.Y.); heqz9923@mails.jlu.edu.cn (Q.H.); gaoyl2321@mails.jlu.edu.cn (Y.G.); 2 Edmond H. Fischer Signal Transduction Laboratory, School of Life Sciences, Jilin University, Changchun 130012, China

**Keywords:** mulberry leaf compounds, Alzheimer’s disease, type 2 diabetes mellitus, network pharmacology, molecular dynamics simulation

## Abstract

Recently, studies have reported a correlation that individuals with diabetes show an increased risk of developing Alzheimer’s disease (AD). Mulberry leaves, serving as both a traditional medicinal herb and a food source, exhibit significant hypoglycemic and antioxidative properties. The flavonoid compounds in mulberry leaf offer therapeutic effects for relieving diabetic symptoms and providing neuroprotection. However, the mechanisms of this effect have not been fully elucidated. This investigation aimed to investigate the combined effects of specific mulberry leaf flavonoids (kaempferol, quercetin, rhamnocitrin, tetramethoxyluteolin, and norartocarpetin) on both type 2 diabetes mellitus (T2DM) and AD. Additionally, the role of the gut microbiota in these two diseases’ treatment was studied. Using network pharmacology, we investigated the potential mechanisms of flavonoids in mulberry leaves, combined with gut microbiota, in combating AD and T2DM. In addition, we identified protein tyrosine phosphatase 1B (PTP1B) as a key target for kaempferol in these two diseases. Molecular docking and molecular dynamics simulations showed that kaempferol has the potential to inhibit PTP1B for indirect treatment of AD, which was proven by measuring the IC_50_ of kaempferol (279.23 μM). The cell experiment also confirmed the dose-dependent effect of kaempferol on the phosphorylation of total cellular protein in HepG2 cells. This research supports the concept of food–medicine homology and broadens the range of medical treatments for diabetes and AD, highlighting the prospect of integrating traditional herbal remedies with modern medical research.

## 1. Introduction

Alzheimer’s disease (AD) is a prevalent neurodegenerative disease. It causes 60–80% of dementia cases and notably impacts the daily lives and well-being of the elderly [1,2,3,4,5]. Diabetes mellitus, as a chronic metabolic disease, is one of the most important public health threats in the 21st century [6,7,8]. Type 2 diabetes mellitus (T2DM) primarily arises from cells that inadequately respond to insulin, resulting in elevated levels of blood sugar [9,10]. As the most common type, T2DM accounts for 90–95% of diagnosed diabetes cases and poses a substantial health burden [11,12]. Furthermore, compelling evidence suggests a shared pathophysiology between T2DM and AD [13,14,15,16]. Mechanistically, impaired glucose absorption in neurons disrupts energy production, exacerbating cognitive impairment. Recent studies have underscored the pivotal role of insulin in brain function, with the brain as an important target of insulin. Dysfunction in insulin signaling among individuals with T2DM can lead to the overactivation of glycogen synthase kinase-3, resulting in elevated tau phosphorylation, modifications of tau, and the degeneration of neurofibrils. Additionally, prolonged hyperglycemia may further worsen the blockage of blood flow to the brain [14]. Based on the association between the two diseases, some theories have even suggested labeling AD “Type 3 Diabetes” [17,18,19]. This association involves intricate pathways including insulin resistance, insulin growth factor signaling, inflammation, oxidative stress, the glycogen synthase kinase 3β (GSK3β) signaling mechanism, amyloid beta formation, neurofibrillary tangle formation, and altered acetylcholine esterase activity [19]. These pathways collectively contribute to the progression of both T2DM and AD, underscoring the complex interplay between metabolic dysfunction and cognitive decline.

Mulberry leaves refer to the dried leaves obtained from the *Morus alba* plant. Renowned as a traditional herbal remedy, they boast a spectrum of beneficial properties including antibacterial and anti-inflammatory effects, blood sugar regulation, blood pressure reduction, lipid-lowering effects, antioxidant properties, and even potential anticancer benefits [20,21,22,23,24,25,26,27,28]. These leaves can be used in culinary applications as well as tea preparation. In Japan, the mulberry leaf tea is affectionately referred to as “longevity tea”. Importantly, given its lack of theophylline and caffeine, mulberry leaf tea is a favorable choice for individuals suffering from stomach discomfort or seeking a soothing drink before bedtime. The main active ingredients of mulberry leaf include flavonoids, alkaloids, phytosterols, γ-aminobutyric acid, mulberry leaf polysaccharides, and more [22]. Notably, the flavonoid quercetin has been proven to be effective in improving diabetes symptoms and exhibiting neuroprotective effects [29,30,31,32]. Our cluster analysis aimed to identify structural analogs of quercetin in mulberry leaf that may play a role in intervening in AD.

Additionally, existing research has confirmed a causal relationship between gut microbiota and cognitive deficits in AD [33,34]. The potential mechanism involves bidirectional regulation through neuro-endocrine-immune network pathways connecting the enteric nervous system with the central nervous system [35,36]. The combined use of gut microbiota and quercetin structural analogs holds promising potential for intervening in AD.

Network pharmacology amalgamates bioinformatics and pharmacology to comprehensively comprehend how drugs function within biological systems. It anticipates the multifaceted impacts of drugs, identifies novel drug targets, and amalgamates protein-compound/disease-gene networks to elucidate drug mechanisms [37,38,39]. We combine computer-assisted screening and network pharmacology to explore how flavonoids (notably kaempferol), in conjunction with the gut microbiota, intervene in T2DM and AD. A pivotal shared target implicated in both conditions, protein tyrosine phosphatase 1B (PTP1B), was pinpointed [40,41,42]. PTP1B is a tyrosine phosphatase whose main function is to remove phosphate groups from proteins. It plays an important role in regulating various metabolisms such as diabetes, obesity, and cancer-related pathways [43,44]. Molecular docking and molecular dynamics simulation assessed the interaction between the target PTP1B and flavonoid molecules derived from mulberry leaf. Subsequently, experimental validation confirmed kaempferol’s ability to inhibit the PTP1B target, highlighting its potential for intervening in diabetes and AD [40,41,42].

Overall, this study endeavors to shed light on the specific targets and pathways involved in leveraging quercetin analogs from mulberry leaves for the prevention and mitigation of T2DM and AD. The findings suggest that kaempferol, in collaboration with the gut microbiota, notably through PTP1B modulation, exhibits potential for intervention in both T2DM and AD. This research reinforces the notion that food and medicine share common roots, broadening the spectrum of therapeutic options for diabetes and AD, and highlighting the importance of integrating traditional herbal remedies with modern medical research.

## 2. Results

**Figure 1:**
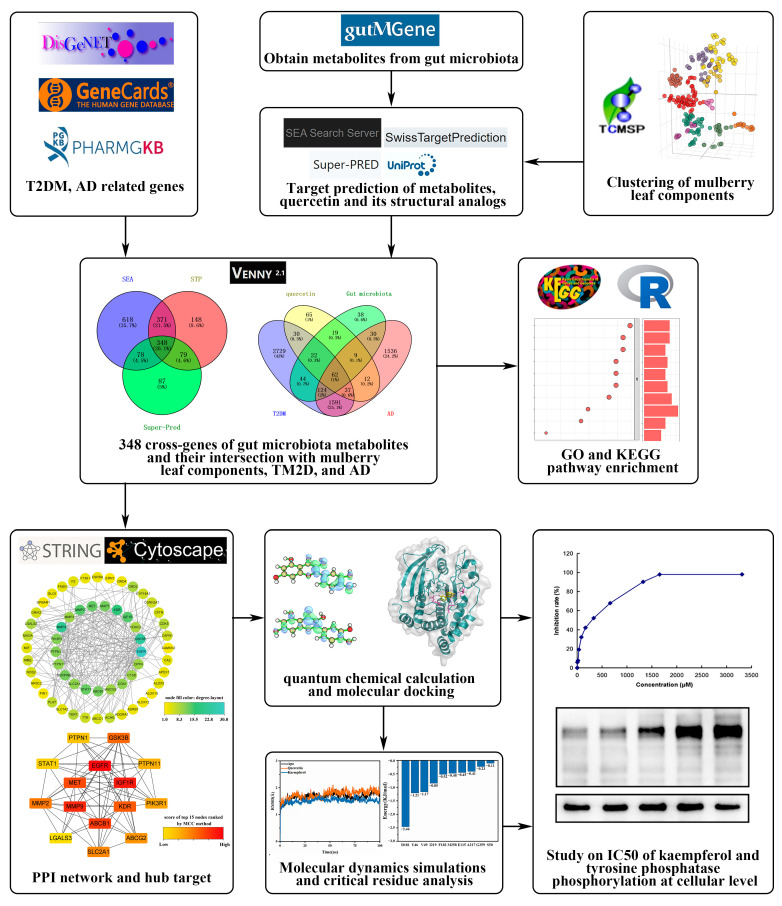
The workflow of the network pharmacology approaches revealed the core targets and molecular mechanisms of quercetin and its structural analogs combined with gut microbiota to treat TM2D and AD. In quantum chemistry computational plots, green indicates positive orbital phases, while blue indicates negative orbital phases.

Figure 1 shows the process of our work. To begin, this study employed a cluster analysis to group together the structural analogs of quercetin found in mulberry leaf. Subsequently, genetic targets sourced from diverse online databases were compiled to identify potential targets associated with specific flavonoids of mulberry leaf (kaempferol, quercetin, rhamnocitrin, tetramethoxyluteolin, and norartocarpetin), gut microbiota, AD, and T2DM. Then, we analyzed the mechanisms of flavonoids in mulberry leaves against AD and T2DM through GO and KEGG enrichment as well as a PPI network analysis. Molecular docking, molecular dynamics simulations, and experiments were used to investigate the inhibitory effect of kaempferol on PTP1B.

### 2.1. Cluster Analysis of Mulberry Leaf Components

**Figure 2:**
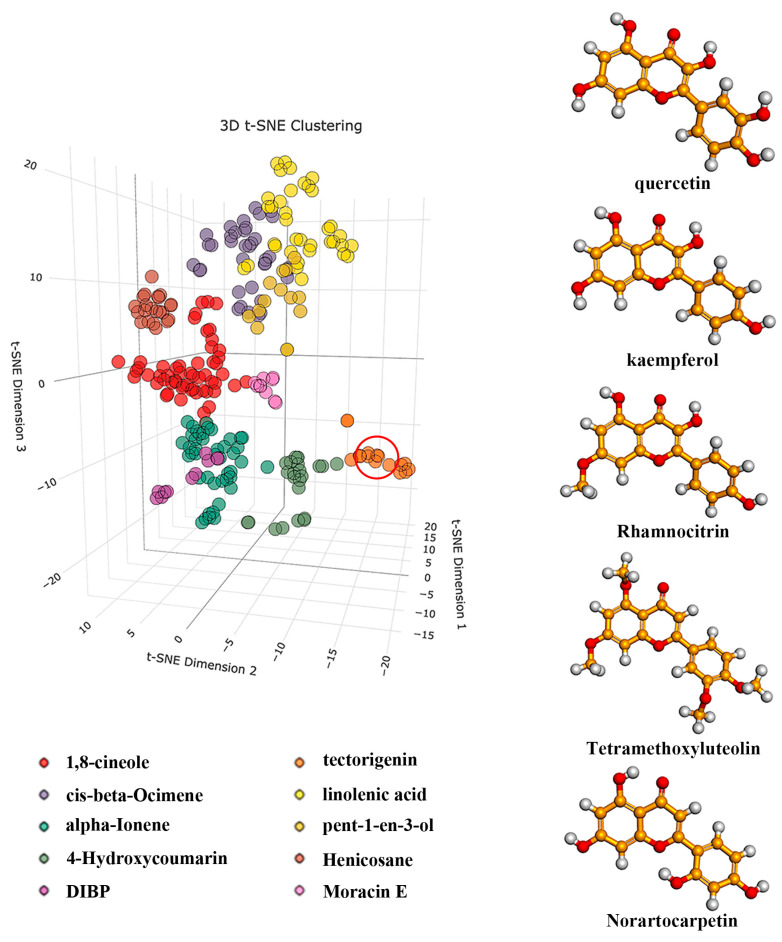
The left part of the figure depicts the coordinates of ten categories of mulberry leaf components. Within the red circle are the spatial positions of quercetin and its four closest compounds. The right part illustrates the structures of quercetin and the four closest compounds.

The SMILES formula of the active small molecules, acquired by searching mulberry leaf components in the TCMSP database, was utilized for t-SNE clustering. Based on the structural similarity of small molecules, 269 components of mulberry leaves were divided into ten categories. The molecule closest to the centroid coordinates within each category was designated as the representative molecule, and we named each category based on these representative molecules (Figure 2) (Table S1). The results of t-SNE clustering showed that the molecules kaempferol, quercetin, rhamnocitrin, tetramethoxyluteolin, and norartocarpetin of mulberry leaf flavonoids are in the same category (Henicosane). Table 1 shows their spatial coordinates.

### 2.2. Targets of Mulberry Leaf Components Combined with Gut Microbiota to Intervene in AD and T2DM

**Figure 3:**
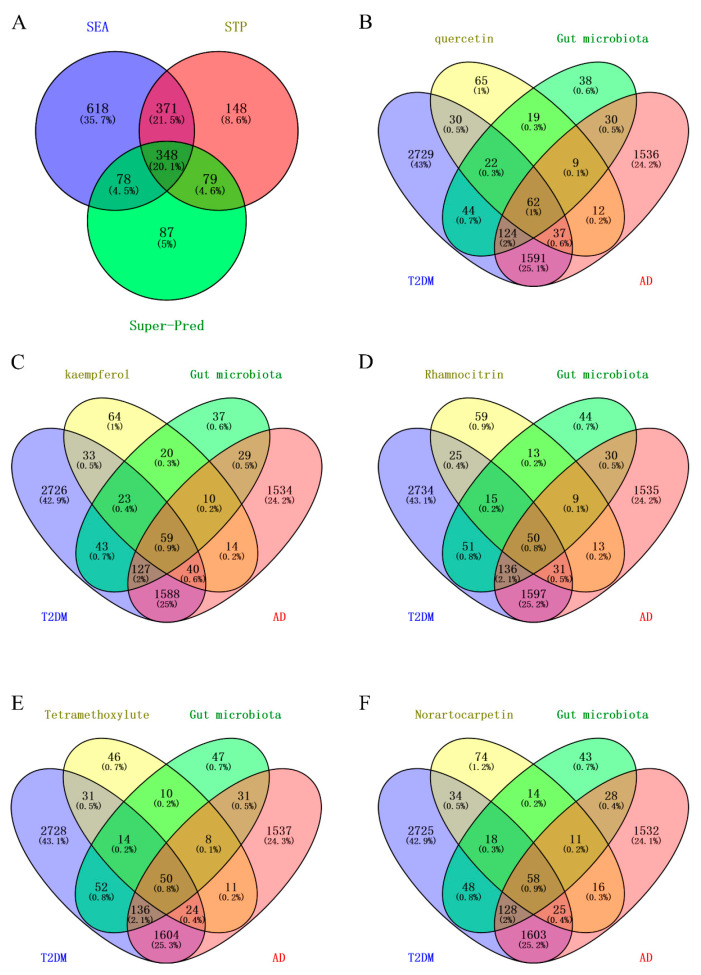
(**A**) Venn diagram showing that there are 348 common targets predicted by SEA, STP, and Super-Pred for gut microbiota secondary metabolites. (**B**) The number of core targets of quercetin is 62. (**C**) The number of core targets of kaempferol is 59. (**D**) The number of core targets of rhamnocitrin is 50. (**E**) The number of core targets of tetramethoxyluteolin is 50. (**F**) The number of core targets of norartocarpetin is 58.

For gut microbial metabolites, 348 common targets were identified across Super-pred [45], SwissTargetPrediction (STP) [46], and SEA [47] (Figure 3A).

Moreover, quercetin shares 62 common targets with gut microbiota, AD, and T2DM (Figure 3B). Likewise, kaempferol, rhamnocitrin, tetramethoxyluteolin, and norartocarpetin exhibit 59, 50, 50, and 58 common targets, respectively (Figure 3C–F). These shared targets are defined as core targets of the above five mulberry components (Table S2). The core targets indicate the potential targets for each component, in collaboration with gut microbiota, to intervene in AD and T2DM.

### 2.3. Construction of PPI Networks and Top 15 Targets’ Screening

**Figure 4:**
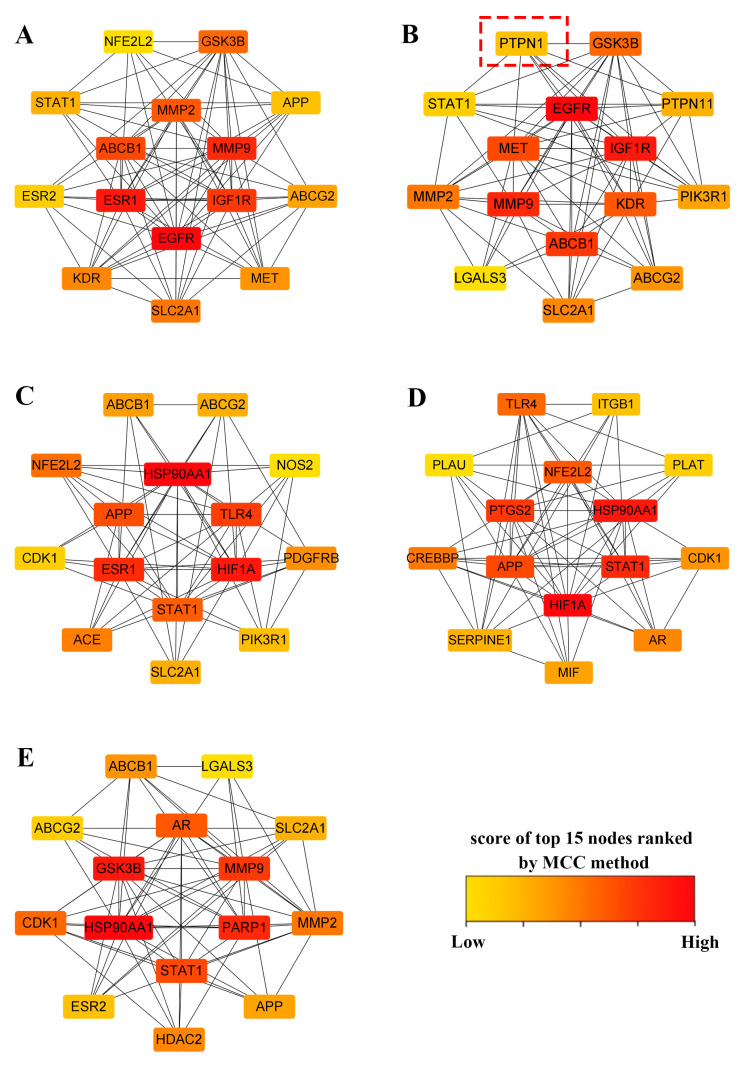
(**A**) Top 15 targets of quercetin. (**B**) Top 15 targets of kaempferol. PTPN1 (PTP1B) is pointed by the red box. (**C**) Top 15 targets of rhamnocitrin. (**D**) Top 15 targets of tetramethoxyluteolin. (**E**) Top 15 targets of norartocarpetin.

The core targets of mulberry leaf components were input into the STRING [48] database to construct the PPI networks (Figure S1). Table 2 shows the nodes, edges, average node degree, and average local clustering coefficient of the PPI network. Using cytoHubba [49] and applying the topology analysis method of Maximal Clique Centrality (MCC), a functionally related protein network of mulberry leaf components combined with intestinal microbial intervention in AD and T2DM was created. Scores estimating relationships between nodes and edges were obtained using the MCC algorithm, where higher scores indicate more significant correlations between genes and the two diseases. We screened the top 15 targets with the highest scores for each active ingredient (Figure 4).

### 2.4. GO Gene Enrichment and KEGG Pathway Analysis

A gene ontology (GO) enrichment analysis is the prevailing method for assessing the enrichment of gene ontology terms within gene collections. It typically categorizes genes into three main levels: the cellular component (CC), biological process (BP), and molecular function (MF). The Kyoto Encyclopedia of Genes and Genomes (KEGG) pathway enrichment analysis primarily focuses on delineating the roles of genes within metabolic and signaling pathways. By examining the enrichment of pathways within gene sets, we can gain a comprehensive understanding of the functions and regulatory mechanisms of these genes within organisms.

**Figure 5:**
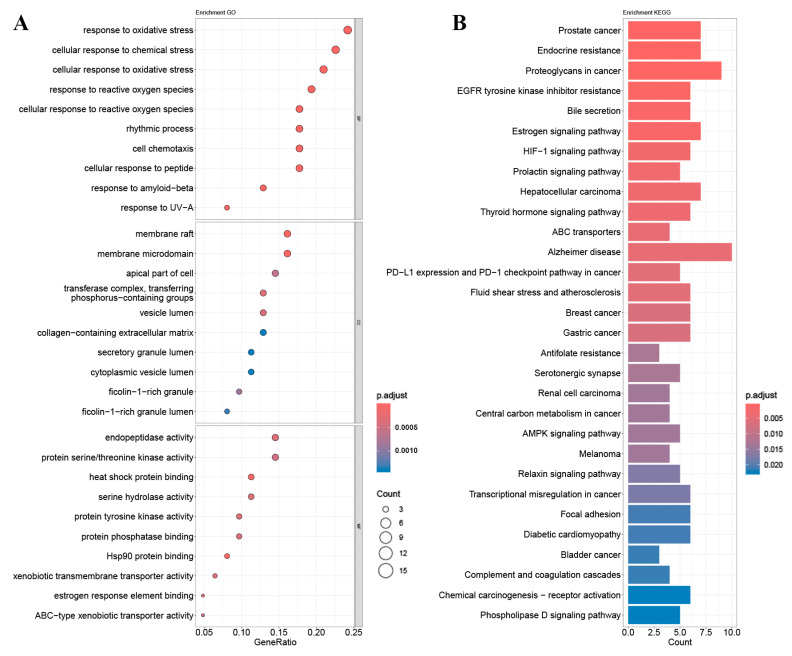
Results of (**A**) GO and (**B**) KEGG pathway enrichment analysis based on the core targets of quercetin.

**Figure 6:**
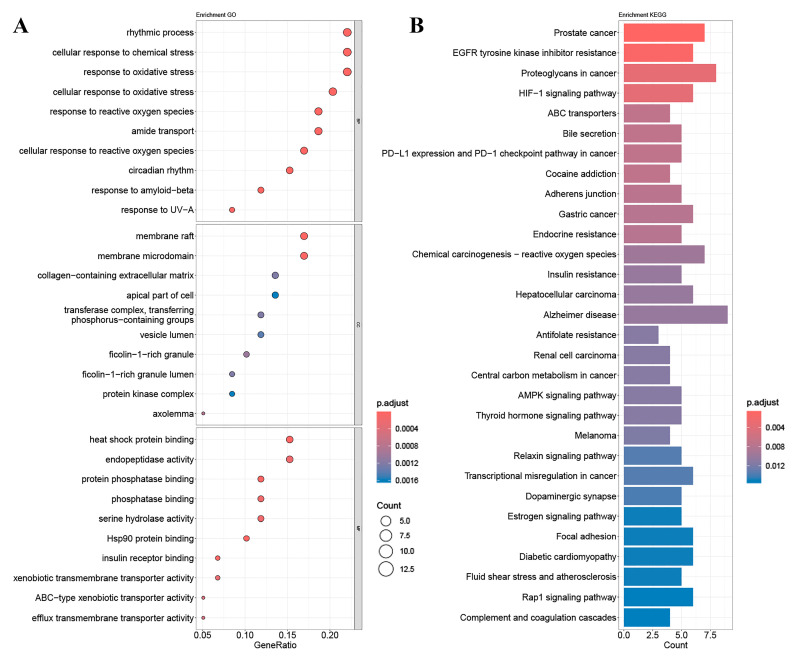
Results of (**A**) GO and (**B**) KEGG pathway enrichment analysis based on the core targets of kaempferol.

**Figure 7:**
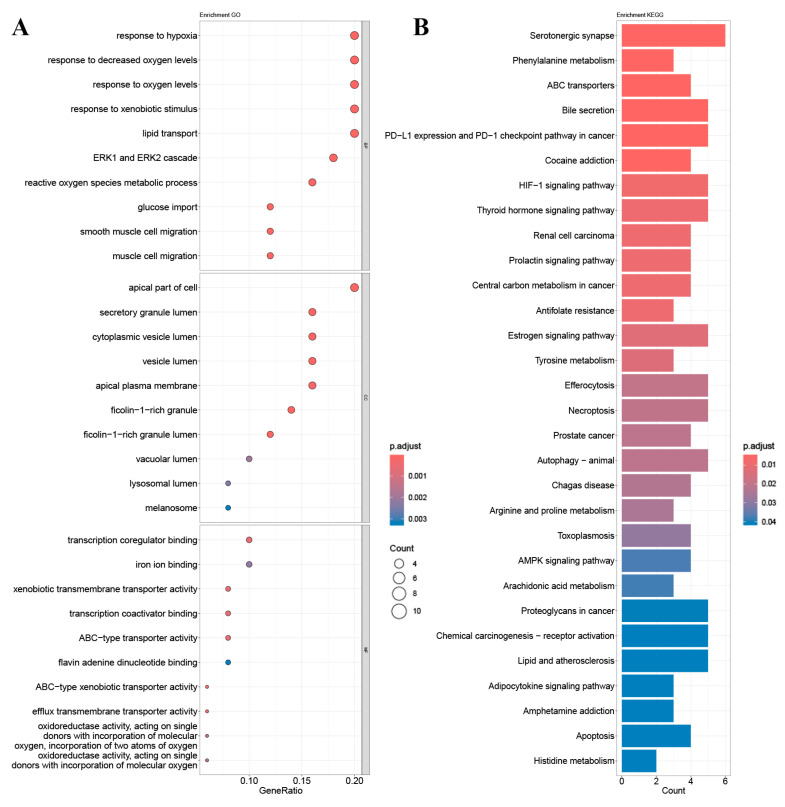
Results of (**A**) GO and (**B**) KEGG pathway enrichment analysis based on the core genes of rhamnocitrin.

**Figure 8:**
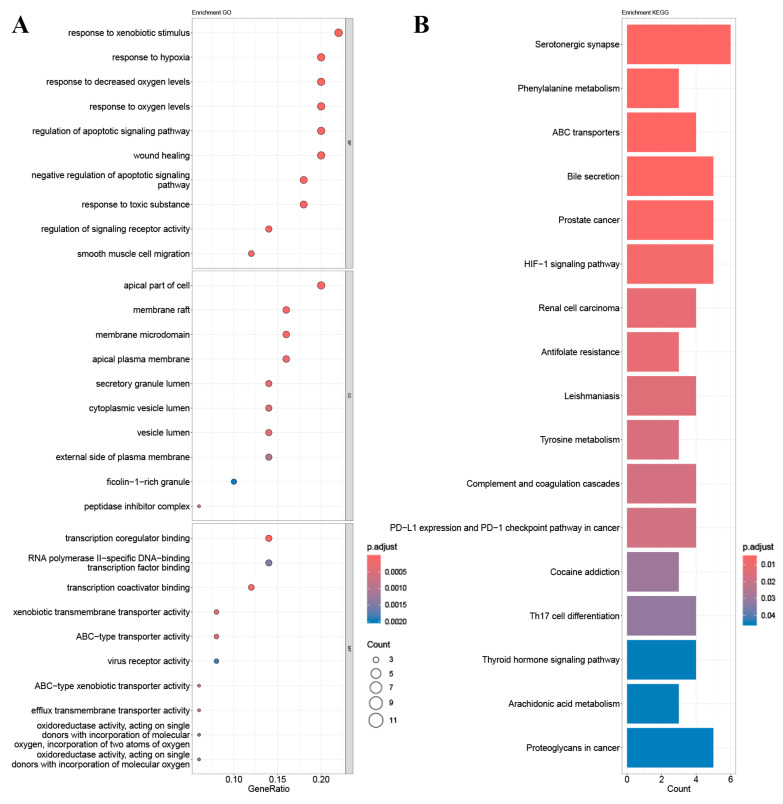
Results of (**A**) GO and (**B**) KEGG pathway enrichment analysis based on the core targets of tetramethoxyluteolin.

**Figure 9:**
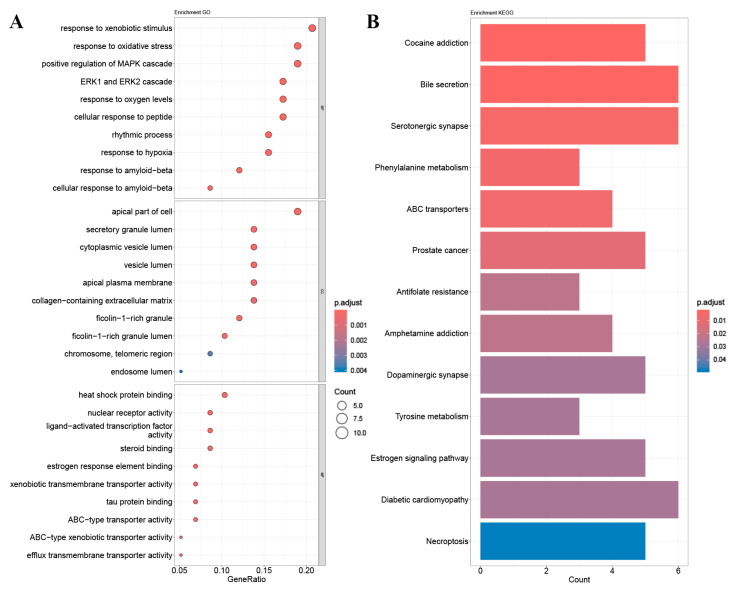
Results of (**A**) GO and (**B**) KEGG pathway enrichment analysis based on the core targets of norartocarpetin.

Enrichment analysis of GO and KEGG pathways was performed using the core targets of mulberry leaf components. The calculated data were demonstrated as bubble charts and histograms derived from GO and KEGG (Figure 5, Figure 6, Figure 7, Figure 8 and Figure 9).

The core targets of five flavonoids, including quercetin, kaempferol, norartocarpetin, rhamnocitrin, and tetramethoxyluteolin, are associated with various biological processes. Quercetin, kaempferol, and norartocarpetin are linked to the response to oxidative stress, the rhythmic process, and amyloid-beta. Meanwhile, rhamnocitrin, norartocarpetin, and tetramethoxyluteolin are associated with the response to the xenobiotic stimulus, response to oxygen levels, and response to hypoxia. In summary, flavonoid compounds present in mulberry leaves exhibit promising neuroprotective strategies by combating oxidative stress, regulating circadian rhythms, and mitigating the effects of amyloid-beta. Additionally, they play a role in cellular defense mechanisms against chemical and reactive oxygen species challenges.

Among the core targets enriched in cellular components, the five flavonoids—quercetin, kaempferol, norartocarpetin, rhamnocitrin, and tetramethoxyluteolin—are all associated with the apical part of the cell, vesicle lumen, and ficolin-1-rich granule. In these cell locations, flavonoids from mulberry leaves mainly act to produce their effects. Furthermore, except for tetramethoxyluteolin, all five demonstrate an association with the ficolin-1-rich granule lumen. Moreover, with the exception of kaempferol, the remaining four are linked to the secretory granule lumen and cytoplasmic vesicle lumen. The ficolin-1-rich granule lumen, secretory granule lumen, and cytoplasmic vesicle lumen are also potential sites where flavonoids in mulberry leaves may manifest their influence.

The molecular functions of core targets include a variety of activities related to transporter functions and protein binding. Specifically, five flavonoids are associated with xenobiotic transmembrane transporter activity and ABC-type xenobiotic transporter activity. Additionally, four of these compounds, excluding quercetin, are linked to efflux transmembrane transporter activity. Furthermore, kaempferol, norartocarpetin, and quercetin are associated with heat shock protein binding, while norartocarpetin, rhamnocitrin, and tetramethoxyluteolin demonstrate involvement in ABC-type transporter activity. In summary, the anti-AD and -T2DM effects of flavonoids in mulberry leaves may be associated with xenobiotic transmembrane, ABC-type, ABC-type xenobiotic, efflux transmembrane transporter, and heat shock protein binding activities.

The enriched KEGG pathways of quercetin, kaempferol, rhamnocitrin, tetramethoxyluteolin, and norartocarpetin were similar and they were all related to ABC transporters, bile secretion, prostate cancer, and antifolate resistance; quercetin, kaempferol, rhamnocitrin, and tetramethoxyluteolin were all related to the HIF-1 signaling pathway and renal cell carcinoma; quercetin, rhamnocitrin, tetramethoxyluteolin, and norartocarpetin were all related to the serotonergic synapse.

### 2.5. Quantum Chemical Calculation

**Figure 10:**
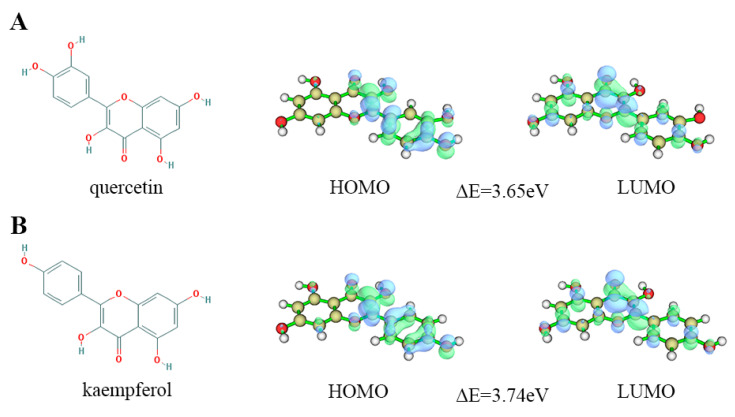
Molecular structure and HOMO-LUMO orbital diagram of (**A**) quercetin and (**B**) kaempferol, green indicates orbital phases are positive, while blue indicates orbital phases are negative.

The Gaussian quantification calculation utilizing the B3LYP/6- 31G* method was employed to analyze five components of mulberry leaf. The calculations produced the following HOMO-LUMO orbital results: (1) quercetin—HOMO orbital energy = −5.52 eV and LUMO orbital energy = −1.86 eV, with an energy gap of 3.65 eV; (2) kaempferol—HOMO orbital energy = −5.55 eV and LUMO orbital energy = −1.81 eV, with an energy gap of 3.74 eV (Figure 10). The calculation results of the HOMO-LUMO orbital energies of rhamnocitrin, tetramethoxyluteolin, and norartocarpetin are shown in Figure S2.

### 2.6. Molecular Docking

**Figure 11:**
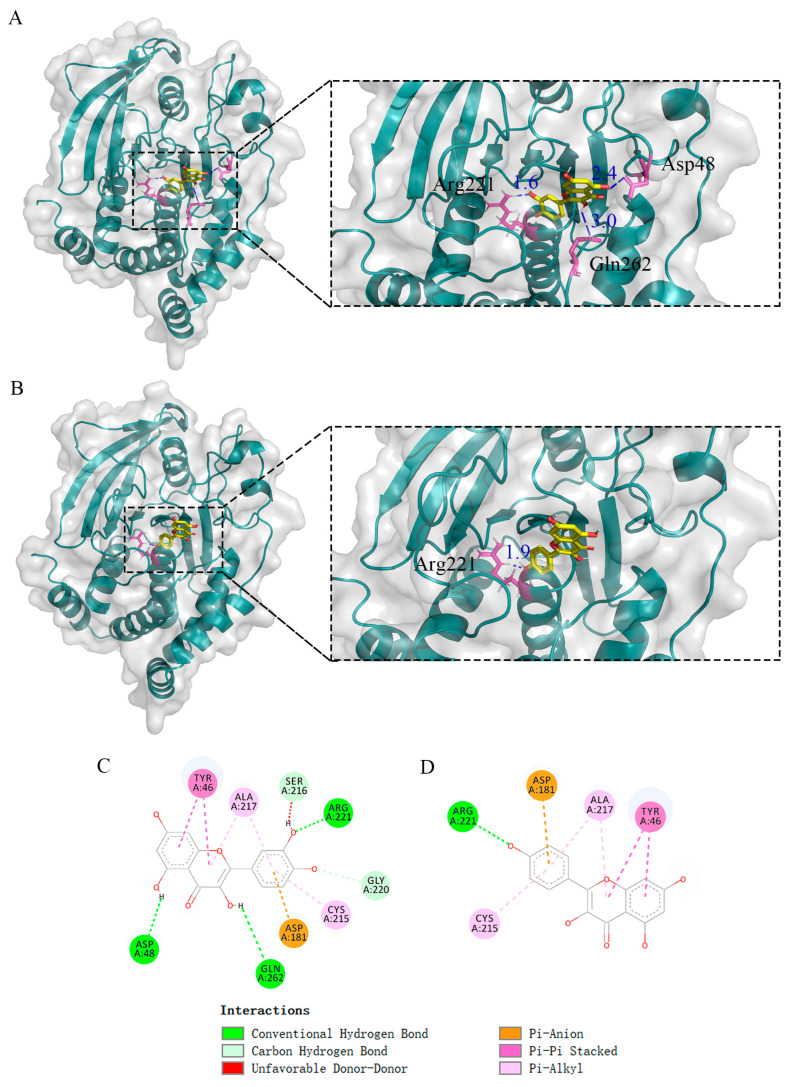
(**A**) Molecular docking indicated the possible binding sites between PTP1B and quercetin. (**B**) Molecular docking indicated the possible binding sites between PTP1B and kaempferol. (**C**) PTP1B and quercetin docking 2D diagram. (**D**) PTP1B and kaempferol docking 2D diagram. For quercetin and kaempferol, the key residues for target genes (PTP1B) may be Arg221, Asp181, Ala217, Cys215, and Tyr46.

Within the PPI network associated with kaempferol, PTPN1, as one of the top 15 hub genes, aroused our interest. PTPN1 encodes for PTP1B, which is a ubiquitous prototype non-receptor tyrosine phosphatase that plays a dephosphorylation role in the KEGG pathways that are enriched by kaempferol. These pathways involve the adherens junction, insulin resistance, and chemical carcinogenesis—reactive oxygen species (Figure 6). Experiments have confirmed that quercetin is an effective inhibitor of PTP1B [50]. Therefore, we speculate that kaempferol, as a structural analog of quercetin, may play a similar role to quercetin and can be combined with gut microbiota to intervene in AD and T2DM. We performed molecular docking of kaempferol and PTP1B, with quercetin serving as a control. For PTP1B, we utilized the 2VEV structure available in the Protein Data Bank (PDB). The docking box was defined by the original ligand position, setting the dimensions to 40, 50, and 40 grids in x, y, and z dimensions, respectively, with a grid of 0.375 Å. The docking results showed that kaempferol is situated at the periphery of the pocket formed by three folds (Figure 11B). The conventional hydrogen bonds between kaempferol and the residues of PTP1B were Arg221, and the benzene ring in Tyr46 interacts with the benzene ring in kaempferol (Figure 11D). The position and interactions of kaempferol closely resemble those of quercetin (Figure 11A,C). Molecular docking revealed an optimal conformational affinity of −7.40 kcal/mol for kaempferol and −7.60 kcal/mol for quercetin. Additionally, Figure S3 shows our molecular docking results using the updated and higher-resolution 8SKL structure and they are similar to 2VEV.

### 2.7. Molecular Dynamics Simulations

**Figure 12:**
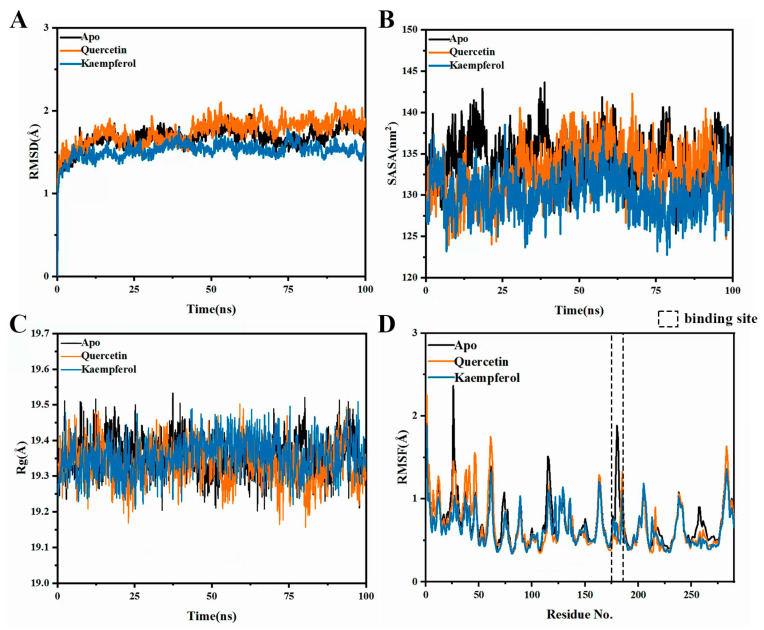
Molecular dynamics simulation of quercetin and kaempferol binding to PTP1B. (**A**) RMSD. (**B**) SASA. (**C**) R_g_. (**D**) RMSF.

To acquire more intricate and comprehensive insights into molecular behavior, we conducted MD simulations. There were three systems in total, including PTP1B combined with kaempferol named Kaempferol, PTP1B combined with quercetin named Quercetin, and PTP1B named Apo. Firstly, the root mean square deviation (RMSD) of the atomic positions within the Cα atoms was computed to evaluate the stability throughout the MD simulations. After binding with kaempferol, PTP1B exhibited a reduced RMSD compared to the unbound protein, indicating an overall increase in stability (Figure 12A); secondly, the reduction in solvent-accessible surface area (SASA) indicated a tighter binding between PTP1B and kaempferol, leading to a decrease in the protein’s surface area and a more enclosed conformation. This change may suggest a potential tightening of the binding pocket (Figure 12B); furthermore, the radius of gyration (R_g_) characterized the overall compactness of a protein structure by measuring the square root of the average distance of atoms from the center of mass within the molecule. After binding with kaempferol, R_g_ demonstrated enhanced stability with reduced fluctuations, indicating a more compact and denser molecular structure (Figure 12C); finally, to investigate the mobility of receptor residues, calculations were performed on the Root Mean Square Fluctuation (RMSF) of backbone atoms. The RMSF results revealed that the flexibility of the binding site (Asp181 and Phe182) decreased upon kaempferol binding (Figure 12D). Throughout the MD simulations, both kaempferol and the control, quercetin, showcased remarkably similar effects on PTP1B. This similarity strongly suggested that kaempferol, similar to quercetin, may indeed act as a PTP1B inhibitor.

**Figure 13:**
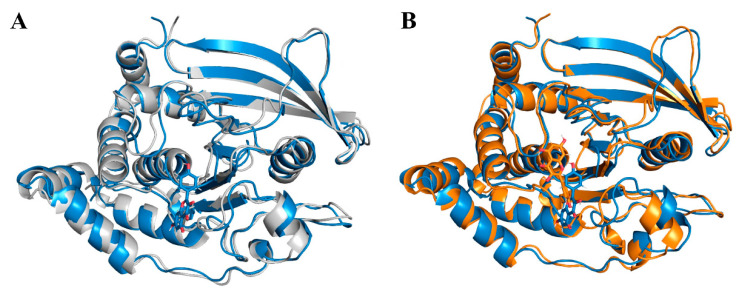
Comparison of representative conformations. (**A**) Kaempferol (blue) and Apo (gray). (**B**) Kaempferol (blue) and Quercetin (orange).

Representative conformations were obtained through K-means clustering. Figure 13A compares the representative conformation of kaempferol bound to PTP1B with the representative conformation of apo. Figure 13B compares the representative conformation of kaempferol bound to PTP1B with the representative conformation of quercetin bound to PTP1B.

The observed alteration in PTP1B suggested a degree of conformational change, with the active pocket indeed contracting toward the ligand, which is consistent with our previous SASA analysis.

**Figure 14:**
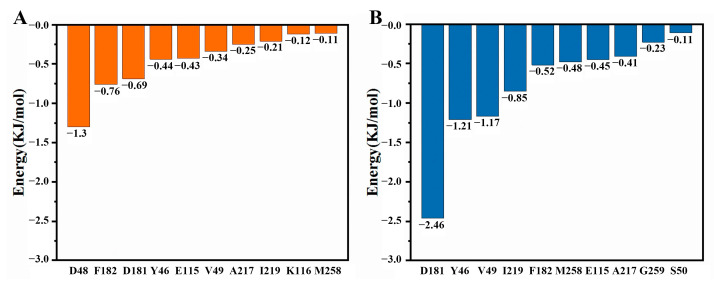
Key residues for quercetin and kaempferol binding to PTP1B. (**A**) Quercetin. (**B**) Kaempferol.

The MM-PBSA results for the interaction between kaempferol and PTP1B are shown in Table 3. Figure 14B displays the key residues for kaempferol binding to PTP1B. Although the individual contribution of each residue varies, both kaempferol and quercetin exhibit a notable similarity in their key residues. Specifically, Asp181, Tyr46, Val49, Ile219, Phe182, Met258, Glu115, and Ala217 are identified as common key residues between them. In addition, in the binding of kaempferol and PTP1B, Gly259 and Ser50 emerge as crucial residues. In summary, we found that more residues are involved in the binding of kaempferol and PTP1B as a complement to the key residues obtained from molecular docking, providing a complementary perspective.

**Figure 15:**
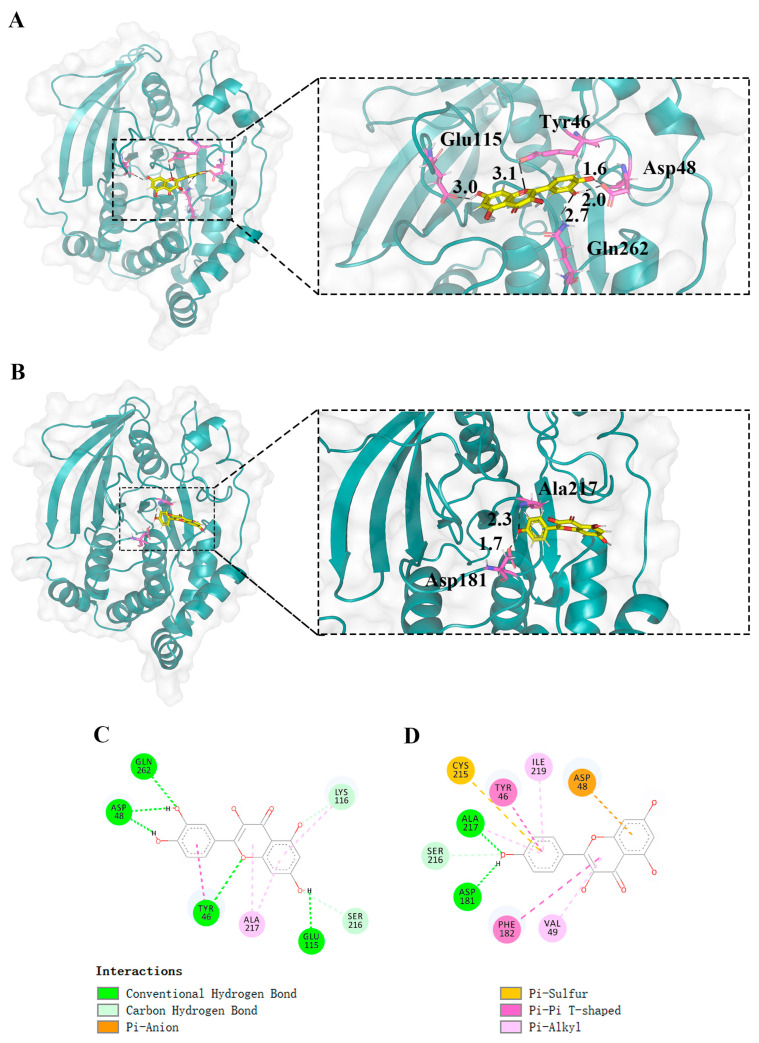
Docking results after MD simulation. (**A**) Three-dimensional docking visualization of PTP1B with quercetin. (**B**) Three-dimensional docking visualization of PTP1B with kaempferol. (**C**) Two-dimensional docking interaction diagram of PTP1B with quercetin. (**D**) Two-dimensional docking interaction diagram of PTP1B with kaempferol.

Figure 15A shows the position of kaempferol relative to PTP1B after MD simulation. Figure 15D shows the interaction between kaempferol and PTP1B. The conventional hydrogen bonds between kaempferol and residues of PTP1B were Ala217 and Asp181. The benzene ring in Tyr46 and Phe182 interacts with the benzene ring in kaempferol. After MD simulation, the hydrogen bond distances between kaempferol and PTP1B became shorter, with increased interactions. We obtained more stable conformations of the binding between kaempferol and PTP1B.

### 2.8. Half Maximal Inhibitory Concentration (IC_50_) and Tyrosine Phosphorylation of Kaempferol

**Figure 16:**
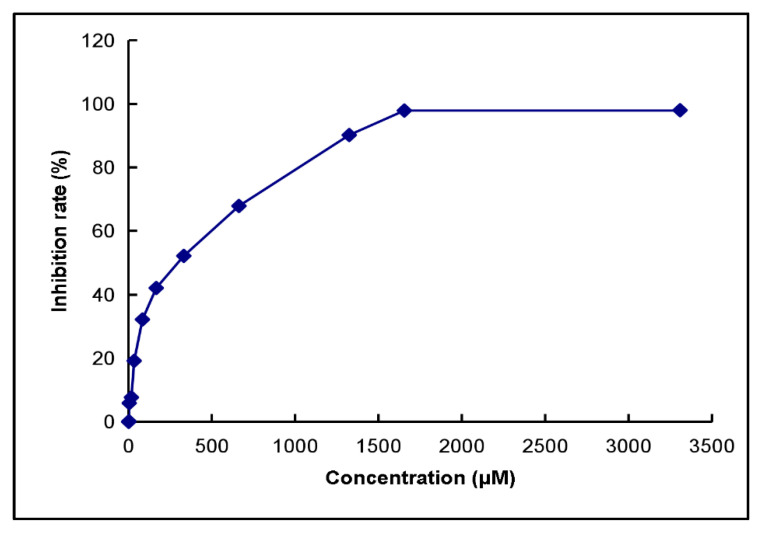
IC_50_ of kaempferol on PTP1B.

The half-inhibitory concentration of PTP1B was tested. The inhibitory constant of kaempferol on PTP1B is 279.23 μM (Figure 16) (Table S3).

**Figure 17:**
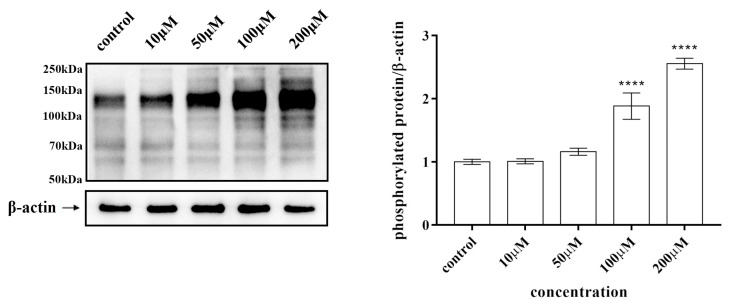
Tyrosine phosphorylation of cellular proteins induced by different concentrations of kaempferol in HepG2 cells. Quantitative analysis of phosphorylated protein/β-actin in the control. Data presented as mean ± SD. **** *p* < 0.0001.

After stimulating HepG2 cells with varying concentrations of kaempferol for 30 min, Western blot results revealed a dose-dependent increase in the tyrosine phosphorylation level of total cellular protein (Figure 17). Treatment with kaempferol at 100 μM and 200 μM significantly elevated the total protein tyrosine phosphorylation level in HepG2 cells (Figure 17). These findings indirectly validate the inhibitory capacity of kaempferol on PTP1B.

### 2.9. Prediction of ADMET Properties

Using ADMETLAB 2.0, predictions were made for the absorption, distribution, metabolism, excretion, and toxicity of kaempferol. The results indicated favorable intestinal absorption for kaempferol, as evidenced by the Caco-2 Permeability (−4.974) and Human Intestinal Absorption (0.008) scores (detailed assessment criteria in Figure S5, point 3). Caco-2 Permeability is a measure of a compound’s permeability across human intestinal epithelial cells (Caco-2 cells), and a Caco-2 Permeability higher than −5.15 Log unit suggests good intestinal absorption. The literature indicates that the lipophilic nature of kaempferol enables its absorption in the small intestine through mechanisms such as passive diffusion, facilitated diffusion, or active transport. When individuals absorb 14.9 mg/d of kaempferol from the intestine, plasma concentrations reach 16.69 ng/mL [51]. Distribution can be assessed using the Volume Distribution index, with values falling within the range of 0.04–20 L/kg considered optimal. Kaempferol exhibited uniform distribution in tissues and plasma, with a Volume Distribution of 0.522. Reported works indicate that kaempferol is metabolized in the liver and small intestine, with metabolites being absorbed into the systemic circulation, distributed to various tissues, and ultimately excreted via feces or urine [52]. Furthermore, the database predicted a low likelihood of kaempferol crossing the blood–brain barrier (Blood–Brain Barrier Penetration = 0.009; detailed assessment criteria in Figure S5, point 4). In terms of metabolism, kaempferol is more likely to inhibit CYP1A2, CYP2D6, and CYP3A4 enzymes (assessment criteria in Figure S5, point 5). The interaction of kaempferol with the CYP450 enzyme may increase its bioavailability [53]. For excretion, kaempferol’s Clearance was predicted to be 6.868 mL/min/kg, indicating a moderate level. Studies have shown that 1.9–2.5% of ingested kaempferol is lost in the urine [52]. Additionally, kaempferol’s half-life was predicted to be greater than 3 h (T1/2 = 0.905; detailed assessment criteria in Figure S5, point 6). Regarding toxicity, kaempferol was predicted to have low Rat Oral Acute Toxicity (0.156), indicating good safety (detailed assessment criteria in Figure S5, point 7).

Compared to quercetin, kaempferol exhibited better drug-likeness, improved intestinal absorption, and higher bioavailability (detailed assessment criteria in QED, Caco-2 Permeability, and F20% in Figures S4 and S5).

## 3. Discussion

In this study, we employed a network pharmacology approach to explore how flavonoids in mulberry leaf, combined with gut microbiota, may intervene in AD and T2DM.

Due to the proven effectiveness of the flavonoid quercetin in improving diabetes symptoms and exhibiting neuroprotective effects, we first conducted a clustering analysis to identify analogs of quercetin, including kaempferol, rhamnocitrin, tetramethoxyluteolin, and norartocarpetin.

Following the construction of PPI networks and conducting a GO and KEGG enrichment analysis, we identified the key component kaempferol and the potential therapeutic target PTP1B. In terms of structure, quercetin is most similar to kaempferol (closest in the clustering analysis), prompting further exploration and analysis. Kaempferol shares 59 common targets with gut microbiota, AD, and T2DM. Utilizing the topological properties of the PPI network (analyzed through cytoHubba), we ranked the targets. PTPN1 held the 13th position, placing it among the top 15 hub genes. That demonstrated its close interaction with other proteins related to AD and T2DM. The PTPN1-encoded PTP1B is a well-established protein crucial in intervening in diabetes, and it is also in the predicted targets associated with AD [40,41,42]. We hypothesize that PTPN1 is a key target through which kaempferol indirectly influences AD. The KEGG enrichment analysis of core targets of kaempferol confirmed our hypothesis, revealing that PTPN1 participates in the significantly enriched (*p* value < 0.01, q value < 0.01) pathways, including the adherens junction, insulin resistance, and chemical carcinogenesis—reactive oxygen species—where it plays a role in the upstream dephosphorylation of IRS-1, MET, and β-Catenin, regulating downstream pathways. Moreover, based on the PPI network, PTPN1 has direct interactions with GSK3B, PIK3R1, and CAPN1 in the Alzheimer’s disease pathway. These may be mechanisms through which PTPN1 indirectly intervenes in AD.

In previous studies conducted in our laboratory, the inhibitory effect of quercetin on PTP1B was confirmed [45]. Due to the structural similarity between kaempferol and quercetin, we inferred that kaempferol could also inhibit PTP1B. Subsequently, molecular docking and MD simulations were performed for kaempferol and PTP1B, and the inhibitory effect of kaempferol on PTP1B was experimentally validated.

It is noteworthy that the position of kaempferol after our MD simulations closely resembles the location of the small-molecule inhibitor of PTP1B (ABBV-CLS-484) in the study by Shuwei Liang et al. [54]. The planar naphthalene core of the ABBV-CLS-484 is situated between the protein exterior’s Phe182 and the hydrophobic surface formed by residues Tyr46, Val49, Ile219, and Ala217. The naphthol moiety of the inhibitor engages in hydrogen bonding with Asp181, while the fluorine atom is in proximity to Gln262. These residues significantly overlap with the binding pocket and crucial interaction residues identified in our MD simulations. Furthermore, in the study by Brent Douty et al., the inhibitor occupies a pocket formed by residues Arg47, Asp48, Val49, Ile219, Gln262, and Phe182, similar to the position of kaempferol in our investigation [55]. This substantiates the accuracy and reliability of our MD results.

Additionally, the results obtained from ADMETLAB 2.0 have revealed the drawbacks of kaempferol as a drug and provided directions for improvement (Figure S5). Kaempferol exhibited low solubility values, as indicated by its Fsp3 value (the number of sp3 hybridized carbons/total carbon count) of zero. Furthermore, the MCE-18 value of kaempferol, being less than 45, suggests a poor balance between its pharmacological activity and chemical properties. The Plasma Protein Binding (PPB) of kaempferol is 95.496%, suggesting a potentially low therapeutic index. Modifying the structure or employing suitable pharmaceutic preparation could enhance the bioavailability and therapeutic index of kaempferol. Furthermore, toxicity prediction for kaempferol indicates the potential for Drug-Induced Liver Injury. This toxicity can be mitigated by adjusting the dosage and employing structural modifications in the drug development process.

Compared with other network pharmacology studies exploring the therapeutic mechanisms of traditional herbal medicines [56,57], we provided a more comprehensive analysis by integrating multiple databases and bioinformatics tools, as well as through molecular dynamics and experimental validation. This facilitated a more comprehensive understanding of how mulberry leaf components impact AD through pharmacological mechanisms. Specifically, our research considered the potential interplay between AD, T2DM, and the microbiome, revealing that kaempferol, a structural analog of quercetin, might possess indirect intervention capabilities in AD. Moreover, our study moved beyond predictions, conducting real biological assays to determine the half-inhibitory concentration (IC_50_) of kaempferol on PTP1B and tyrosine phosphorylation levels, providing empirical evidence supporting our computational predictions. Our research is mutually confirmed through a combination of dry and wet experiments. It enhanced the reliability and accuracy of our research, ensuring that our findings hold biological significance.

Despite these advantages, there are some limitations in our findings. For the metabolites obtained from the gutMGene database, we did not consider whether their substrates were ingested by the human body. Some metabolites that did not have a SMILES type in PubChem were also ignored by us. At the same time, our focus was solely on the potential collaborative targets of kaempferol and gut microbiota in addressing AD and T2DM. We did not account for the potential influence of kaempferol on the gut microbiota itself, despite such an influence potentially existing [58]. Additionally, our focus primarily centered on studying the impact of kaempferol on the PTP1B target protein, omitting an in-depth exploration of other quercetin analogs and alternative targets. Furthermore, although there are existing reports on the interaction mechanisms among AD, T2DM, and intestinal microorganisms, their precise relationship remains unclear. A clearer understanding of the interaction mechanisms between AD and other diseases, as well as AD and gut microbiota, could significantly advance research on the indirect effects of herbal ingredients on AD.

Overall, this study provided valuable insights into the potential pharmacological mechanisms and therapeutic effects of mulberry leaf components, especially kaempferol, combined with gut microbiota, to intervene in AD and T2DM. By analyzing the connections between these targets, we can help uncover and address limitations and challenges in treating AD. Future research can continue to advance our understanding of the complex interactions between traditional herbal medicine and modern scientific methods, ultimately helping the development of safe and efficacious treatments, either direct or indirect, for AD and its associated conditions.

## 4. Materials and Methods

### 4.1. Acquisition and Cluster Analysis of Components in Mulberry Leaf

Mulberry leaf components were obtained through the TCMSP database (https://old.tcmsp-e.com/tcmsp.php, accessed on 23 October 2023) [59], and a total of 270 components were found. Clustering was implemented through the R programming language, and we used several R packages, including rcdk, dplyr, rcdklibs, Rtsne, stats, ggplot2, and plotly. The process began by translating SMILES notations into binary fingerprint vectors and subsequently reducing data dimensions using t-SNE for three-dimensional visualization. Following this, the data were clustered to uncover groups or clusters sharing similarities among molecules. Ultimately, the clusters were visualized in a 3D plot using plotly for an in-depth analysis, and the clustered data were saved.

### 4.2. Prediction of AD-Related Targets and T2DM-Related Targets

First, we screened for potential T2DM target genes from the DisGeNET (https://www.disgenet.org/, accessed on 30 October 2023), GeneCards (https://www.genecards.org/, accessed on 28 October 2023), and PharmGKB [60] (https://www.pharmgkb.org/, accessed on 30 October 2023). We identified 3134 potential targets in DisGeNET databases, 2950 potential targets with a relevance > 0.1 in GeneCards, and 55 potential targets in PharmGKB. After performing a union operation on the potential targets from three databases, 4639 unique potential targets were obtained. The same method was used to search for potential AD targets (we accessed DisGeNET, GeneCards, and PharmGKB on 28 October 2023), identifying 3401 results.

### 4.3. Prediction of Targets of Quercetin and Its Structural Analogs

We screened Super-Pred (https://prediction.charite.de/subpages/target_prediction.php, accessed on 30 October 2023), SwissTargetPrediction (http://swisstargetprediction.ch/, accessed on 30 October 2023), and SEA (http://sea.bkslab.org/, accessed on 30 October 2023) databases for potential targets on which quercetin and its structural analogs may act. For quercetin, we identified 132 potential targets in Super-Pred databases, 86 potential targets with a Probability > 0.4 in STP, and 147 potential targets in SEA. After performing a union operation on the potential targets from three databases, 256 unique potential targets were obtained. Similarly, for kaempferol, we obtained 263 targets; for rhamnocitrin, we obtained 215 targets; for tetramethoxyluteolin, we obtained 194 targets; and for norartocarpetin, we obtained 250 targets.

### 4.4. Prediction of Gut Microbiota Metabolite Targets

The gutMGene database was used to obtain metabolites of gut microbiota (http://bio-annotation.cn/gutmgene/, accessed on 12 November 2023) [61], and a total of 208 metabolites were obtained. PubChem was used to obtain SMILES formulas of metabolites, resulting in 184 SMILES formulas. The resulting SMILES formulas were entered into Super-Pred (accessed on 21 November 2023), STP (accessed on 21 November 2023), and SEA (accessed on 22 November 2023) to predict its potential targets. The obtained targets were screened based on the human gene names in the swiss-prot data set in the UniProt database (https://www.uniprot.org/, accessed on 30 October 2023) [62], and 592 targets were obtained in Super-Pred, 946 targets were obtained in STP, and 1415 targets with Probability > 0 were obtained in SEA.

### 4.5. Acquisition Targets for Mulberry Leaf Ingredients Combined with Gut Microbiota to Intervene in AD and T2DM

For accuracy, we utilized the online tool VENNY2.1 (https://bioinfogp.cnb.csic.es/tools/venny/index.html, accessed on 28 November 2023) to assess the overlap of gut microbiota metabolite targets among Super-pred, STP, and SEA. These targets, alongside predicted mulberry leaf component targets, AD-related targets, and T2DM-related targets, were comprehensively analyzed using VENNY2.1.

### 4.6. Construction of the Protein–Protein Interaction (PPI) Network

The construction of the PPI network relied on data sourced from the STRING database (http://string-db.org/, accessed on 29 November 2023) to evaluate potential interactions among the identified hub targets. The visual representation of these interactions was achieved using Cytoscape (version 3.9.1). Subsequently, the PPI network’s topological properties were analyzed, leading to the identification of the top 15 hub genes through Cytoscape’s analytical tool—cytoHubba.

### 4.7. GO and KEGG Enrichment Analysis

We employed several R packages—clusterProfiler, AnnotationHub, org.Hs.eg, enrichplot, pathview, dplyr, and ggplot2—for conducting enrichment analyses on gene ontology (GO) [63,64] biological processes and Kyoto Encyclopedia of Genes and Genomes (KEGG) based on core target data. Utilizing a significance threshold of *p* = 0.01 and q = 0.01, we retrieved GO information from org.Hs.eg.Db in Bioconductor. The outcomes are presented through bar charts and bubble charts to offer a comprehensive visualization of the final results.

### 4.8. Quantum Chemical Calculation

Gaussian09W and GaussView 5.0 [65] were used to perform quantitative calculations on the structures of quercetin and its analogs. The Method of Gaussian Calculation was B3LYP of DFT in the Ground State, and we made the Basis Set 6-31G*. The visual HOMO-LUMO orbitals’ diagrams of the quantitative calculation were drawn through Multiwfn [66].

### 4.9. Molecular Docking

We delved deeper into the gene target XDH, one of the top 15 hub genes identified in the PPI network associated with kaempferol. The gene encodes for protein tyrosine phosphatase 1B (PTP1B). Kaempferol and quercetin were selected for molecular docking with PTP1B (PDB code: 2VEV, including the N-terminal Glu2-Glu300 part of PTP1B), the latter being a confirmed inhibitor of PTP1B, serving as a control. To prepare the receptor, we utilized Discovery Studio 2019 for dehydration and hydrogenation. Subsequently, AutoDock Vina 1.2.0 [67] was employed for the docking process, and the results of molecular docking were visualized using Pymol 2.5.7 [68] and Discovery Studio. Figure S6 displays the results of our docking validation.

### 4.10. Molecular Dynamics Simulations

Using AMBER 16’s PMEMD engine and the FF14SB AMBER force field [69], we conducted MD simulations on our systems. We employed the TIP3P water model and periodic boundary conditions to prevent edge effects. After setting the appropriate system distance and neutralizing ions, we conducted steps involving energy minimization, gradual heating, and equilibration. The simulations, spanning 100 ns, operated under the isobaric–isothermal ensemble (NPT) conditions, featuring randomized seeding, constant pressure of 1 bar (maintained by the Berendsen barostat), a pressure-coupling constant of 2 ps, a temperature of 300 K, and a Langevin thermostat [70] with a collision frequency of 1 ps. The analyses of MD were conducted utilizing CPPTRAJ [71], and the obtaining of representative conformations was also conducted utilizing the K-means cluster method of the CPPTRAJ module.

Similar to our previous work [72], we employed the molecular mechanics/Poisson–Boltzmann surface area (MM/PBSA) method to investigate the binding affinity between kaempferol and PTP1B. The binding free energy (Δ*G_bind_*) is represented by the following formula:

(1) $$\Delta G_{bind}=\Delta H-T\Delta S$$

(2) $$\Delta H=\Delta E_{MM}+\Delta G_{sol}$$

(3) $$\Delta E_{MM}=\Delta E_{ele}+\Delta E_{vdW}+\Delta E_{\mathrm{int}}$$

(4) $$\Delta G_{sol}=\Delta G_{pb}+\Delta G_{np}$$

In the above formula, Δ*E_MM_* represents the gas-phase energy, Δ*G_sol_* represents the solvation-free energy, Δ*E_ele_* represents electrostatic energy, Δ*E_vdW_* represents van der Waals energy, Δ*E_int_* represents internal energy, Δ*G_pb_* represents the polar solvation-free energy, and Δ*G_np_* represents non-polar solvation-free energy.

We conducted calculations every 2 ns, ultimately extracting 50 snapshots from the final trajectory for MM/PBSA calculations.

### 4.11. IC_50_ and Tyrosine Phosphorylation of Kaempferol

To investigate kaempferol’s impact on PTP1B activity, we determined its IC_50_.

Following the methodology outlined in previous literature, the catalytic domain (Met1-Glu298) of PTP1B (ΔPTP1B) was synthesized using BL21 bacteria harboring the PT7-ΔPTP1B recombinant plasmid, subsequently isolated and purified [73].

Utilizing p-Nitrophenyl Phosphate (PNPP) as a non-specific substrate for protein tyrosine phosphatase, ΔPTP1B catalyzes its breakdown into p-nitrophenol. The quantification of protein tyrosine phosphatase activity was achieved by measuring the mole count of p-nitrophenol generated.

Initially, we diluted the kaempferol sample to varying concentrations. By monitoring the resulting changes in the production of the reaction product (p-nitrophenol) across different kaempferol concentrations, we gauged the inhibitory effect of kaempferol on PTP1B, constructing a concentration-dependent inhibition curve. The IC_50_ value was derived from this curve. Our experiment utilized a 100 µL system, encompassing diverse kaempferol concentrations (16.25, 32.5, 65, 125, 250, 500, 1000, and 2000 μM). Table 4 shows the remaining system components.

Following a 15 min incubation at 37 °C, the reaction was halted by adding 100 µL of 0.1 M NaHCO_3_ (PH ≈ 8.4). The experiment quantified the p-nitrophenol yield through absorbance measurement at a 405 nm wavelength, following the methodology outlined in previous literature. A blank group lacking PTP1B served as the control for comparison.

To detect the tyrosine phosphorylation effect of kaempferol, HepG2 cells were cultured in a growth medium supplemented with 10% fetal calf serum until reaching approximately 90% confluence. Subsequently, the cells were treated with varying concentrations of kaempferol (10, 50, 100, and 200 μM). After a 30 min incubation period, the cells were exposed to a solution comprising 25 mM glycerophosphate (pH 7.3), 5 mM EDTA, 2 mM EGTA, 5 mM mercaptoethanol, 1% Triton X-100, 0.1 M NaCl, and a cocktail of protease and phosphatase inhibitors. Following this, protein extraction was performed by centrifugation at 12,000× *g* for 10 min to isolate the supernatant. Subsequently, proteins were separated using 10% SDS-PAGE gel electrophoresis, transferred onto a polyvinylidene fluoride membrane, and subjected to treatment with a phosphotyrosine antibody (PY99). Enhanced chemiluminescence was employed for detection purposes (Table 5).

### 4.12. Prediction of ADMET Properties

To predict the absorption, distribution, metabolism, excretion, and toxicity of kaempferol and quercetin, we utilized the ADMET Evaluation module of ADMETLAB 2.0 (https://admetmesh.scbdd.com/, accessed on 7 February 2024) [74] by submitting the SMILES formulas of kaempferol and quercetin.

## 5. Conclusions

In this study, we employed a network pharmacology approach to explore how structural analogs of quercetin found in mulberry leaf, especially kaempferol, combined with gut microbiota, may intervene in AD and T2DM. By constructing a PPI network and analyzing the top 15 hub genes, we identified the key component kaempferol and the potential therapeutic target PTP1B. By utilizing a GO enrichment and KEGG pathway analysis, we can delve deeply into comprehending the biological processes and molecular functions associated with core genes. Network pharmacology reveals that quercetin structural analogs combined with gut microbiota have potential functions in the treatment of T2DM and AD.

Molecular docking confirmed the interaction between kaempferol and PTP1B. A further molecular dynamics analysis demonstrated that upon binding with kaempferol, PTP1B exhibited increased stability compared to PTP1B without inhibitors. This binding resulted in a more closed conformation and reduced flexibility at the binding site. Notably, kaempferol exhibited similar effects to the control quercetin.

Experimental findings demonstrate that kaempferol exhibits inhibitory effects on tyrosine phosphatase activity, with an observed IC_50_ value against PTP1B determined at 279.23 µM. Additionally, kaempferol elevates the levels of tyrosine phosphorylation in cells, indicating its potential therapeutic efficacy in T2DM and AD.

To summarize, this study offers a comprehensive comprehension of the pharmacological mechanisms and potential therapeutic impact of mulberry leaf components in intervening in AD. The delineation of crucial components, targets, and signaling pathways holds promise for the development of novel treatments for AD. However, further experimental validation and clinical trials are imperative to confirm the efficacy and safety of kaempferol, potentially paving the way for its utilization as a preventive and therapeutic agent for AD and associated conditions.

## Figures and Tables

**Table 1:** Clustering of components with similar structures in mulberry leaves, the coordinates of quercetin, and the four nearest compounds.

Name	MOL ID	Dimension 1	Dimension 2	Dimension 3
quercetin	MOL000098	13.98971333	−15.4718551	−12.91115122
kaempferol	MOL000422	14.93055973	−15.56046554	−13.2378193
rhamnocitrin	MOL000251	14.27188684	−15.25028232	−13.87688149
tetramethoxyluteolin	MOL007879	14.28316604	−14.20247304	−12.73193365
norartocarpetin	MOL006630	15.14767912	−14.11761894	−13.39243877

**Table 2:** The nodes, edges, average node degree, and average local clustering coefficient of the PPI networks.

Name	Number of Nodes	Number of Edges	Average Node Degree	Avg. Local Clustering Coefficient
quercetin	62	316	10.20	0.46
kaempferol	59	233	7.90	0.42
rhamnocitrin	50	194	7.76	0.52
tetramethoxyluteolin	50	192	7.68	0.55
norartocarpetin	58	227	7.83	0.50

**Table 3:** The MM-PBSA results of kaempferol.

Energy	Value (kcal/mol)
ΔE_vdW_ (van der Waals energy)	−19.06 ± 0.44
ΔE_ele_ (electrostatic energy)	−24.19 ± 1.34
ΔG_gas_ (gas-phase free energy change)	31.89 ± 0.99
ΔG_solv_ (solvation free energy change)	−43.25 ± 1.19
binding free energy	−11.36 ± 0.51

**Table 4:** Remaining components of the 100 µL system.

System Components	Concentration
MOPS-NaOH buffer (pH 7.0)	25 mM
EDTA	1 mM
DTT	1 mM
BSA	1 mg/mL
NaCl	0.1 M
p-NPP	10 mM
PTP1B	40 ng

**Table 5:** Summary table of experimental material sources.

Reagent Name	Manufacturers
BL21 with PT7-ΔPTP1B recombinant plasmid	Laboratory preservation (Fisher Laboratory, College of Life Sciences, Jilin University, Changchun, China)
Kaempferol	Aladdin (Shanghai, China)
HepG2 cell	Nanjing Keygen Company (Nanjing, China)
SDS-PAGE	SIGMA Corporation of America (Ronkonkoma, NY, USA)
Phosphotyrosine antibody (PY99)	Santa Cruz Biotechnology (Santa Cruz, CA, USA) (RRID: AB_628123)
β-Actin antibodies	Santa Cruz Biotechnology (Santa Cruz, CA, USA) (RRID: AB_626632)

## Data Availability

Data are contained within the article and Supplementary Materials.

## Supplementary Materials

The following supporting information can be downloaded at: https://www.mdpi.com/article/10.3390/ijms25074062/s1.
