# Supplementary material for: Mulberry Leaf Compounds and Gut Microbiota in Alzheimer’s Disease and Diabetes: A Study Using Network Pharmacology, Molecular Dynamics Simulation, and Cellular Assays
Source: Int J Mol Sci. 2024 Apr 5;25(7):4062. doi: 10.3390/ijms25074062 (PMC11012793; doi:10.3390/ijms25074062)
Supplement: Supplementary file 1 [file ijms-25-04062-s001.zip › Figure S1_PPI.pdf]

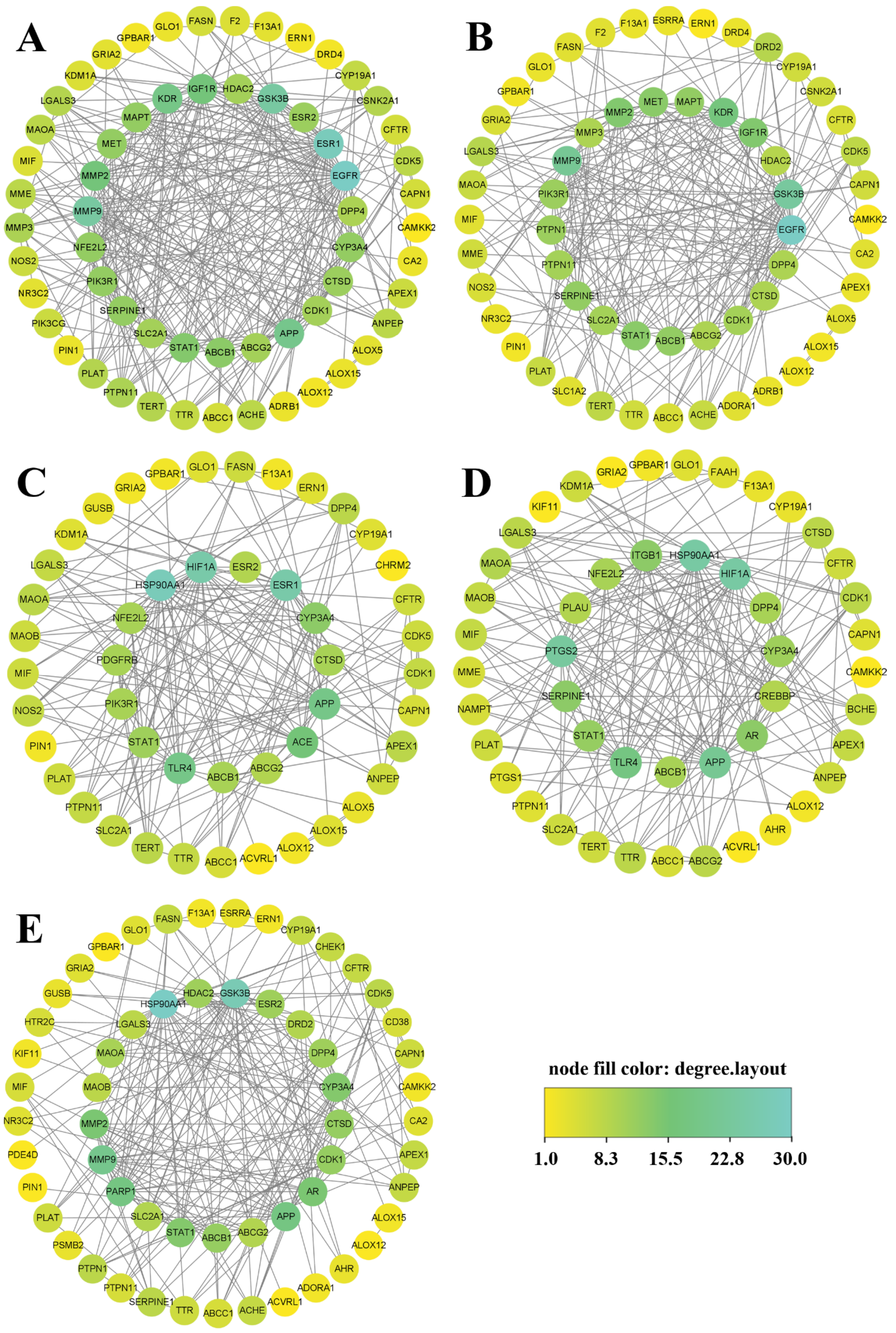

Figure S1. PPI network of (A) quercetin, (B) kaempferol, (C) rhamnocitrin, (D) tetramethoxyluteolin, (E) norartocarpetin.
