# Supplementary material for: Mulberry Leaf Compounds and Gut Microbiota in Alzheimer’s Disease and Diabetes: A Study Using Network Pharmacology, Molecular Dynamics Simulation, and Cellular Assays
Source: Int J Mol Sci. 2024 Apr 5;25(7):4062. doi: 10.3390/ijms25074062 (PMC11012793; doi:10.3390/ijms25074062)
Supplement: Supplementary file 1 [file ijms-25-04062-s001.zip › Figure S2_HOMO-LUMO.pdf]

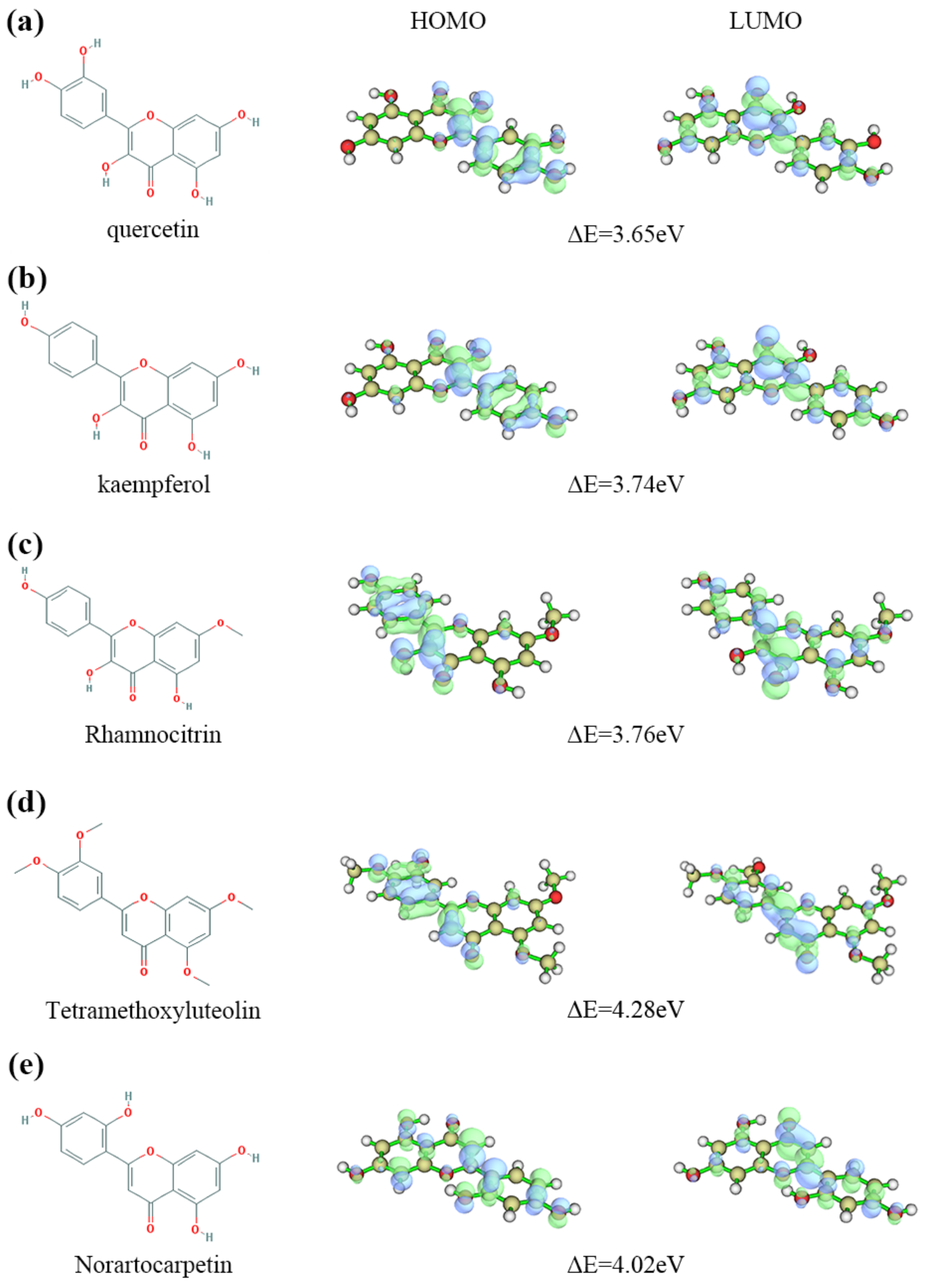

Figure S1. Molecular structure and HOMO-LUMO orbital diagram of (a) quercetin, (b) kaempferol, (c) rhamnocitrin, (d) tetramethoxyluteolin, and (e) norartocarpetin.
