# Supplementary material for: Mulberry Leaf Compounds and Gut Microbiota in Alzheimer’s Disease and Diabetes: A Study Using Network Pharmacology, Molecular Dynamics Simulation, and Cellular Assays
Source: Int J Mol Sci. 2024 Apr 5;25(7):4062. doi: 10.3390/ijms25074062 (PMC11012793; doi:10.3390/ijms25074062)
Supplement: Supplementary file 1 [file ijms-25-04062-s001.zip › Figure S3_8SKL.pdf]

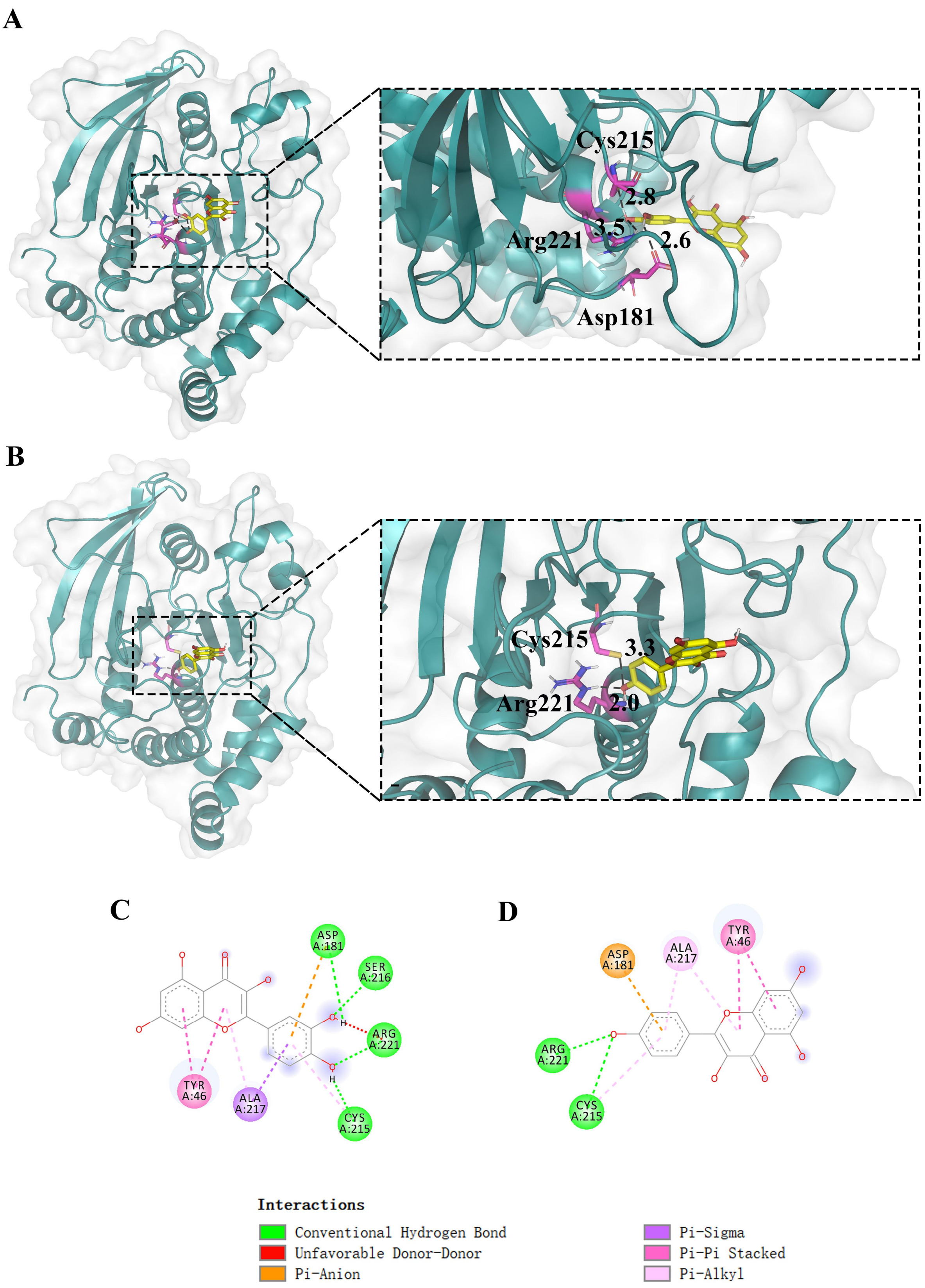

Figure S3. Docking results of the PTP1B（PDB code: 8SKL）with quercetin and kaempferol. (A) 3D docking visualization of PTP1B with quercetin. (B) 3D docking visualization of PTP1B with kaempferol. (C) 2D docking interaction diagram of PTP1B with quercetin. (D) 2D docking interaction diagram of PTP1B with kaempferol. The docking results are similar to the PTP1B structure obtained from PDB code 2VEV.
