# Supplementary material for: Mulberry Leaf Compounds and Gut Microbiota in Alzheimer’s Disease and Diabetes: A Study Using Network Pharmacology, Molecular Dynamics Simulation, and Cellular Assays
Source: Int J Mol Sci. 2024 Apr 5;25(7):4062. doi: 10.3390/ijms25074062 (PMC11012793; doi:10.3390/ijms25074062)
Supplement: Supplementary file 1 [file ijms-25-04062-s001.zip › Figure S5_ADMET of kaempferol.pdf]

ADMETlab 2.0

O=c1c(O)c(-c2ccc(O)cc2)oc2cc(O)cc(O)c12

1. Physicochemical Property

Property	Value	Comment
Molecular Weight	286.05	Contain hydrogen atoms. Optimal:100~600
Volume	273.977	Van der Waals volume
Density	1.044	Density = MW / Volume
nHA	6	Number of hydrogen bond acceptors. Optimal:0~12
nHD	4	Number of hydrogen bond donors. Optimal:0~7
nRot	1	Number of rotatable bonds. Optimal:0~11
nRing	3	Number of rings. Optimal:0~6
MaxRing	10	Number of atoms in the biggest ring. Optimal:0~18
nHet	6	Number of heteroatoms. Optimal:1~15
fChar	0	Formal charge. Optimal:-4 ~4
nRig	18	Number of rigid bonds. Optimal:0~30
Flexibility	0.056	Flexibility = nRot /nRig
Stereo Centers	0	Optimal: ≤ 2
TPSA	111.13	Topological Polar Surface Area. Optimal:0~140
logS	-3.624	Log of the aqueous solubility. Optimal: -4~0.5 log mol/L
logP	2.656	Log of the octanol/water partition coefficient. Optimal: 0~3
logD	2.233	logP at physiological pH 7.4. Optimal: 1~3

2. Medicinal Chemistry

Property	Value	Decision	Comment
QED	0.546	●	■ A measure of drug-likeness based on the concept of desirability;<br>■ Attractive: > 0.67; unattractive: 0.49~0.67; too complex: < 0.34
SAscore	2.375	●	■ Synthetic accessibility score is designed to estimate ease of synthesis of drug-like molecules.<br>■ SAscore ≥ 6, difficult to synthesize; SAscore <6, easy to synthesize
Fsp3	0.0	●	■ The number of sp3 hybridized carbons / total carbon count, correlating with melting point and solubility.<br>■ Fsp^3 ≥0.42 is considered a suitable value.
MCE-18	18.0	●	■ MCE-18 stands for medicinal chemistry evolution.<br>■ MCE-18≥45 is considered a suitable value.

ADMETlab 2.0

NPscore	1.546	-	■ Natural product-likeness score.<br>■ This score is typically in the range from –5 to 5. The higher the score is, the higher the probability is that the molecule is a NP.
Lipinski Rule	Accepted	●	■ MW ≤ 500; logP ≤ 5; Hacc ≤ 10; Hdon ≤ 5<br>■ If two properties are out of range, a poor absorption or permeability is possible, one is acceptable.
Pfizer Rule	Accepted	●	logP > 3; TPSA < 75<br>Compounds with a high log P (>3) and low TPSA (<75) are likely to be toxic.
GSK Rule	Accepted	●	■ MW ≤ 400; logP ≤ 4<br>■ Compounds satisfying the GSK rule may have a more favorable ADMET profile
Golden Triangle	Accepted	●	■ 200 ≤ MW ≤ 50; -2 ≤ logD ≤ 5<br>■ Compounds satisfying the Golden Triangle rule may have a more favorable ADMET profile.
PAINS	0 alerts	-	Pan Assay Interference Compounds, frequent hitters, Alpha-screen artifacts and reactive compound.
ALARM NMR	2 alerts	-	Thiol reactive compounds.
BMS	0 alerts	-	Undesirable, reactive compounds.
Chelator Rule	1 alerts	-	Chelating compounds.

3. Absorption

Property	Value	Decision	Comment
Caco-2 Permeability	-4.974	●	Optimal: higher than -5.15 Log unit
MDCK Permeability	9e-06	●	■ low permeability: < 2 × 10^{-6} cm/s<br>■ medium permeability: 2–20 × 10^{-6} cm/s<br>■ high passive permeability: > 20 × 10^{-6} cm/s
Pgp-inhibitor	0.004	●	■ Category 1: Inhibitor; Category 0: Non-inhibitor;<br>■ The output value is the probability of being Pgp-inhibitor
Pgp-substrate	0.011	●	■ Category 1: substrate; Category 0: Non-substrate;<br>■ The output value is the probability of being Pgp-substrate
HIA	0.008	●	Human Intestinal Absorption<br>Category 1: HIA+(HIA < 30%); Category 0: HIA-(HIA ≥ 30%); The output value is the probability of being HIA+
$F_{20\%}$	0.856	●	■ 20% Bioavailability<br>■ Category 1: $F_{20\%}$+ (bioavailability < 20%); Category 0: $F_{20\%}$- (bioavailability ≥ 20%); The output value is the probability of being $F_{20\%}$+

ADMETlab 2.0

$F_{30\%}$	0.993	🔴	■ 30% Bioavailability<br>■ Category 1: $F_{30\%}$+ (bioavailability < 30%); Category 0: $F_{30\%}$- (bioavailability ≥ 30%); The output value is the probability of being $F_{30\%}$+

4. Distribution

Property	Value	Decision	Comment
PPB	97.86%	🔴	■ Plasma Protein Binding<br>■ Optimal: < 90%. Drugs with high protein-bound may have a low therapeutic index.
VD	0.522	🟢	■ Volume Distribution<br>■ Optimal: 0.04-20L/kg
BBB Penetration	0.009	🟢	■ Blood-Brain Barrier Penetration<br>■ Category 1: BBB+; Category 0: BBB-; The output value is the probability of being BBB+
Fu	4.411%	🔴	■ The fraction unbound in plasms<br>■ Low: <5%; Middle: 5~20%; High: > 20%

5. Metabolism

Property	Value	Comment
CYP1A2 inhibitor	0.972	■ Category 1: Inhibitor; Category 0: Non-inhibitor;<br>■ The output value is the probability of being inhibitor.
CYP1A2 substrate	0.11	■ Category 1: Substrate; Category 0: Non-substrate;<br>■ The output value is the probability of being substrate.
CYP2C19 inhibitor	0.181	■ Category 1: Inhibitor; Category 0: Non-inhibitor;<br>■ The output value is the probability of being inhibitor.
CYP2C19 substrate	0.046	■ Category 1: Substrate; Category 0: Non-substrate;<br>■ The output value is the probability of being substrate.
CYP2C9 inhibitor	0.653	■ Category 1: Inhibitor; Category 0: Non-inhibitor;<br>■ The output value is the probability of being inhibitor.
CYP2C9 substrate	0.867	■ Category 1: Substrate; Category 0: Non-substrate;<br>■ The output value is the probability of being substrate.
CYP2D6 inhibitor	0.722	■ Category 1: Inhibitor; Category 0: Non-inhibitor;<br>■ The output value is the probability of being inhibitor.
CYP2D6 substrate	0.283	■ Category 1: Substrate; Category 0: Non-substrate;<br>■ The output value is the probability of being substrate.
CYP3A4 inhibitor	0.697	■ Category 1: Inhibitor; Category 0: Non-inhibitor;<br>■ The output value is the probability of being inhibitor.
CYP3A4 substrate	0.08	■ Category 1: Substrate; Category 0: Non-substrate;<br>■ The output value is the probability of being substrate.

ADMETlab 2.0

6. Excretion

Property	Value	Decision	Comment
CL	6.868	🟢	■ Clearance<br>■ High: >15 mL/min/kg; moderate: 5-15 mL/min/kg; low: <5 mL/min/kg
$T_{1/2}$	0.905	-	■ Category 1: long half-life ; Category 0: short half-life;<br>■ long half-life: >3h; short half-life: <3h<br>■ The output value is the probability of having long half-life.

7. Toxicity

Property	Value	Decision	Comment
hERG Blockers	0.07	🟢	■ Category 1: active; Category 0: inactive;<br>■ The output value is the probability of being active.
H-HT	0.098	🟢	■ Human Hepatotoxicity<br>■ Category 1: H-HT positive(+); Category 0: H-HT negative(-);<br>■ The output value is the probability of being toxic.
DILI	0.979	🔴	■ Drug Induced Liver Injury.<br>■ Category 1: drugs with a high risk of DILI; Category 0: drugs with no risk of DILI. The output value is the probability of being toxic.
AMES Toxicity	0.672	🟠	■ Category 1: Ames positive(+); Category 0: Ames negative(-);<br>■ The output value is the probability of being toxic.
Rat Oral Acute Toxicity	0.156	🟢	■ Category 0: low-toxicity; Category 1: high-toxicity;<br>■ The output value is the probability of being highly toxic.
FDAMDD	0.109	🟢	■ Maximum Recommended Daily Dose<br>■ Category 1: FDAMDD (+); Category 0: FDAMDD (-)<br>■ The output value is the probability of being positive.
Skin Sensiti zation	0.856	🔴	■ Category 1: Sensitizer; Category 0: Non-sensitizer;<br>■ The output value is the probability of being sensitizer.
Carcinogen city	0.097	🟢	■ Category 1: carcinogens; Category 0: non-carcinogens;<br>■ The output value is the probability of being toxic.
Eye Corrosion	0.009	🟢	■ Category 1: corrosives ; Category 0: noncorrosives<br>■ The output value is the probability of being corrosives.
Eye Irritation	0.929	🔴	■ Category 1: irritants ; Category 0: nonirritants<br>■ The output value is the probability of being irritants.

ADMETlab 2.0

Respiratory Toxicity	0.09	●	■ Category 1: respiratory toxicants; Category 0: respiratory nontoxicants<br>■ The output value is the probability of being toxic.

8. Environmental toxicity

Property	Value	Comment
Bioconcentration Factors	0.986	■ Bioconcentration factors are used for considering secondary poisoning potential and assessing risks to human health via the food chain.<br>■ The unit is –log10[(mg/L)/(1000*MW)]
IGC_{50}	4.386	■ Tetrahymena pyriformis 50 percent growth inhibition concentration<br>■ The unit is –log10[(mg/L)/(1000*MW)]
$LC_{50}FM$	5.223	■ 96-hour fathead minnow 50 percent lethal concentration<br>■ The unit is –log10[(mg/L)/(1000*MW)]
$LC_{50}DM$	5.205	■ 48-hour daphnia magna 50 percent lethal concentration<br>■ The unit is –log10[(mg/L)/(1000*MW)]

9. Tox21 pathway

Property	Value	Decision	Comment
NR-AR	0.008	●	■ Androgen receptor<br>■ Category 1: actives ; Category 0: inactives;<br>■ The output value is the probability of being active.
NR-AR-LBD	0.371	●	■ Androgen receptor ligand-binding domain<br>■ Category 1: actives ; Category 0: inactives;<br>■ The output value is the probability of being active.
NR-AhR	0.967	●	■ Aryl hydrocarbon receptor<br>■ Category 1: actives ; Category 0: inactives;<br>■ The output value is the probability of being active.
NR-Aromatase	0.941	●	■ Category 1: actives ; Category 0: inactives;<br>■ The output value is the probability of being active.
NR-ER	0.959	●	■ Estrogen receptor<br>■ Category 1: actives ; Category 0: inactives;<br>■ The output value is the probability of being active.
NR-ER-LBD	0.985	●	■ Estrogen receptor ligand-binding domain<br>■ Category 1: actives ; Category 0: inactives;<br>■ The output value is the probability of being active.
NR-PPAR-gamma	0.963	●	■ Peroxisome proliferator-activated receptor gamma<br>■ Category 1: actives ; Category 0: inactives;<br>■ The output value is the probability of being active.
SR-ARE	0.873	●	■ Antioxidant response element<br>■ Category 1: actives ; Category 0: inactives;<br>■ The output value is the probability of being active.
SR-ATAD5	0.612	●	■ ATPase family AAA domain-containing protein 5<br>■ Category 1: actives ; Category 0: inactives;<br>■ The output value is the probability of being active.

ADMETlab 2.0

SR-HSE	0.557	●	■ Heat shock factor response element<br>■ Category 1: actives ; Category 0: inactives;<br>■ The output value is the probability of being active.
SR-MMP	0.968	●	■ Mitochondrial membrane potential<br>■ Category 1: actives ; Category 0: inactives;<br>■ The output value is the probability of being active.
SR-p53	0.921	●	■ Category 1: actives ; Category 0: inactives;<br>■ The output value is the probability of being active.

10. Toxicophore Rules

Property	Value	Comment
Acute Toxicity Rule	0 alerts	■ 20 substructures<br>■ acute toxicity during oral administration
Genotoxic Carcinogenicity Rule	0 alerts	■ 117 substructures<br>■ carcinogenicity or mutagenicity
NonGenotoxic Carcinogenicity Rule	0 alerts	■ 23 substructures<br>■ carcinogenicity through nongenotoxic mechanisms
Skin Sensitization Rule	4 alerts	■ 155 substructures<br>■ skin irritation
Aquatic Toxicity Rule	0 alerts	■ 99 substructures<br>■ toxicity to liquid(water)
NonBiodegradable Rule	1 alerts	■ 19 substructures<br>■ non-biodegradable
SureChEMBL Rule	0 alerts	■ 164 substructures<br>■ MedChem unfriendly status

ADMETlab 2.0
