# Supplementary material for: Mulberry Leaf Compounds and Gut Microbiota in Alzheimer’s Disease and Diabetes: A Study Using Network Pharmacology, Molecular Dynamics Simulation, and Cellular Assays
Source: Int J Mol Sci. 2024 Apr 5;25(7):4062. doi: 10.3390/ijms25074062 (PMC11012793; doi:10.3390/ijms25074062)
Supplement: Supplementary file 1 [file ijms-25-04062-s001.zip › Figure S6_docking validation.pdf]

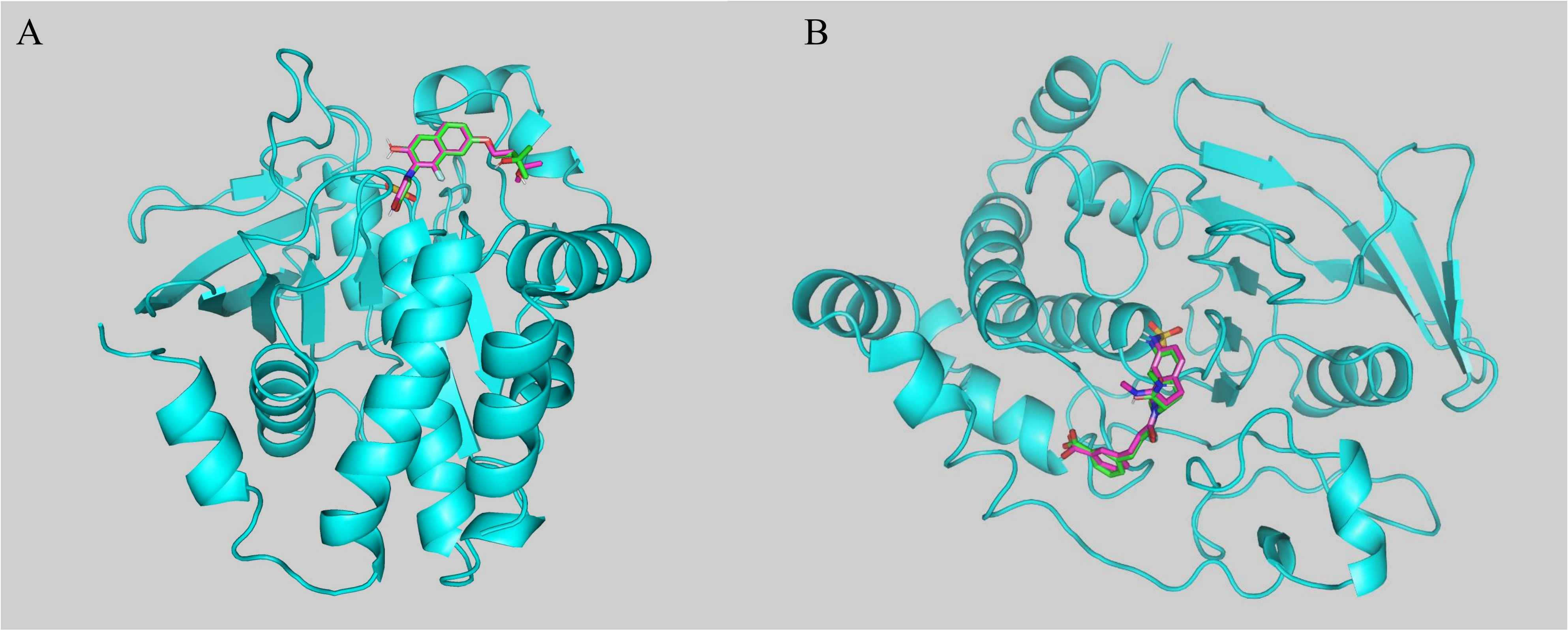

Figure S2. Docking validation of AutoDock Vina 1.2.0. (A) The original ligand position (pink) for PDB code 8SKL and the ligand position after AutoDock docking (green). RMSD = 0.84 Å. (B) The original ligand position (pink) for PDB code 2F71 and the ligand position after AutoDock docking (green). RMSD = 0.87 Å.
